# Supplementary material for: Determinants of penetrance and variable expressivity in monogenic metabolic conditions across 77,184 exomes
Source: Nat Commun. 2021 Jun 9;12:3505. doi: 10.1038/s41467-021-23556-4 (PMC8190084; doi:10.1038/s41467-021-23556-4)
Supplement: Supplementary file 1 — Supplementary Information [file 41467_2021_23556_MOESM1_ESM.pdf]

Determinants of penetrance and variable expressivity in monogenic metabolic conditions across 77,184 exomes

Supplementary Information

Supplementary Tables

1. Summary characteristics of study populations.
2. Counts of clinically significant variants and carriers across conditions.
3. Comparison of Top 1% gePS with interquartile range and monogenic carries.
4. Mean serum LDL values based on ascertainment approach.
5. Impact of polygenic score on trait expressivity in monogenic carriers.
6. Frequency cut-offs used for ClinVar variant curation.

Supplementary Figures

1. Distribution of clinically significant variants across ancestries.
2. Carriers of clinically significant variants in MODY genes show a younger age of diabetes diagnosis compared to the rest of the AMP-T2D-GENES cohorts and UK Biobank population.

List of Consortia Members

Extended Acknowledgements

Supplementary Tables

Supplementary Table 1: Summary characteristics of study populations.

	AMP-T2D-GENES T2D case	AMP-T2D-GENES control	UKB
Male (N)	9,231	9,239	17,732
Female (N)	9,508	10,640	20,834
age	58.20 (9.7)	57.345 (10.65)	58.272 (7.92)
BMI	29.27 (5.95)	27.49 (5.36)	27.36 (4.76)
ldl_mgdl	115.489 (38.26)	130.721 (36.24)	137.089 (33.06)
ldl (med corrected)	134.311 (45.79)	136.394 (39.79)	147.461 (33.06)
hdl_mgdl	46.601 (14.12)	51.775 (16.16)	57.266 (14.92)
tg_mgdl	180.644 (138.31)	139.618 (98.87)	150.605 (86.37)
tg (med corrected)	191.844 (148.60)	142.513 (100.98)	156.935 (92.94)
African American	2,480	2,626	NA
East Asian	2,666	2,533	NA
European	4,713	6,557	38,566
Hispanic	6,126	5,609	NA
Other	3	13	NA
South Asian	2,751	2,541	NA

Supplementary Table 2: Counts of clinically significant variants and carriers across conditions

		AMP T2D-GENES		UK Biobank	
**Condition**	**Genes**	**N_variants**	**N_carriers**	**N_variants**	**N_carriers**
High LDL	***LDLR***, and missense ***APOB***	53	126	37	90
Low LDL	***APOB***, ***PSCK9*** (restricted to LoF)	29	78	40	92
High HDL	***CETP***	15	26	13	23
High triglycerides	***APOA5***, ***LPL***	13	23	10	56
Monogenic obesity	***MC4R***	9	29	13	31
MODY	***GCK***, ***HNF1A***, ***HNF4A***, ***HNF1B***, ***PDX1***	17	22	14	16
MODY Extended	*AKT2*, *KLF11*, *APPL1*, ***ABCC8***, *KCNJ11*, *NEUROD1*, *CEL*, ***INS***	2	2	NA	NA
Lipodystrophy	*AKT2*, ***LMNA,*** *PLIN1*, ***PPARG***	8	8	3	7
Neonatal Diabetes*	***ABCC8***, *GATA4*, *GATA6*, ***INS***, *KCNJ11*, ***HNF1B***	3	3	NA	NA

Note: Bold genes indicate genes that have clinically significant variant carriers
*These variants are incorporated in categories above. The *INS* and *HNF1B* variants were LOF; the *ABCC8* variant has been seen in both neonatal and MODY patients

Supplementary Table 3: Comparison of Top 1% gePS with interquartile range and monogenic carries

	Top 1% of gePS vs Interquartile range (25-75%)		Monogenic carriers vs Top 1% gePS	
**Condition**	**Estimate**	**Pvalue***	**Estimate**	**Pvalue****
Low LDL cholesteol mg/dL (med adj)	-17.23	3.03E-10	-42.87	2.74E-17
Low LDL cholesterol mg/dL (no lipid meds)	-16.01	2.19E-09	-40.29	7.09E-14
High LDL cholesterol mg/dL (med adj)	16.47	2.43E-09	33.72	2.32E-05
High LDL cholesterol mg/dL (no lipid meds)	12.45	3.70E-06	33.28	2.47E-03
High HDL cholesterol mg/dL	6.46	6.82E-10	12.65	2.61E-03
High triglycerides mg/dL	49.57	8.61E-14	51.44	0.04
Obesity kg/m2	1.86	6.96E-09	0.14	0.90
Diabetes Odds Ratio	2.66	4.25E-07	9.95	1.46E-03

*Regression comparing the top 1% to the interquartile range (25-75%) of the gePS. Adjusted for age, sex and 10 PC's. Age in controls restricted to >=60.

**Regression comparing the top 1% of the gePS to carriers in UK Biobank. Adjusted for age, sex and 10 PC's. Age in controls restricted to >=60.

Pvalues two-sided

Supplementary Table 4: Mean serum LDL values based on ascertainment approach.

	Individuals ascertained on serum LDL cholesterol			Individuals not ascertained on LDL cholesterol			
**Condition**	**Total**	**Carriers**	**Carrier LDL***	**Total**	**Carriers**	**Carrier LDL***	**Pvalue****
	**N**	**N**	**Mean (95% CI)**	**N**	**N**	**Mean (95% CI)**	
"High LDL"	249	18	329 (284-375)	19,186	55	198 (178-219)	0.00041
"Low LDL"	253	15	49.2 (43.0-55.4)	19,186	35	77.0 (66.0-88.0)	0.055
**Restricted to shared LDL variants**							
**Condition**	**Total**	**Carriers**	**Carrier LDL***	**Total**	**Carriers**	**Carrier LDL***	**Pvalue*****
"High LDL"	NA	7	321 (235-407)	NA	7	183 (123-244)	0.0116
"Low LDL"	NA	5	54.8 (41.4-68.3)	NA	11	92.0 (64.9-119)	0.0491

*LDL values adjusted for lipid-lowering medication use per methods

**Regression comparing LDL values in carriers ascertained on serum LDL cholesterol to carriers not ascertained. Adjusted for age, sex and 10 PC's

***Regression comparing LDL values in carriers ascertained on serum LDL cholesterol to carriers not ascertained; restricted to shared LDL variants. Adjusted for age, sex and 5 PC's

Pvalues two-sided

Supplementary Table 5: Impact of polygenic score on trait expressivity in monogenic carriers.

A) Effect size of gePS on trait in monogenic variant carriers

Trait	N monogenic carriers	gePS beta*	Pvalue	Beta in direction of increasing expressivity
High LDL	83	-0.88	0.886	no
Low LDL	90	-3.89	0.227	yes
High HDL	20	17.52	0.012	yes
High triglycerides	54	80.57	0.014	yes
Obesity	31	1.48	0.137	yes

*per SD global polygenic risk score (gePS)

Model adjusted for age, sex, PC's; Pvalues two-sided

B) Assessment of interaction of carrier status and gePS

Trait	Variable	beta	Pvalue
Low LDL	CarrierStatus	-56.55	1.87E-52
Low LDL	gePS	-5.55	2.62E-197
Low LDL	CarrierStatus*gePS	2.17	0.54
Low LDL no lipid meds	CarrierStatus	-53.19	9.59E-61
Low LDL no lipid meds	gePS	-5.14	1.17E-185
Low LDL no lipid meds	CarrierStatus*gePS	1.67	0.58
High LDL	CarrierStatus	54.94	5.60E-46
High LDL	gePS	5.58	7.71E-199
High LDL	CarrierStatus*gePS	-4.63	0.2
High LDL no lipid meds	CarrierStatus	47.24	7.13E-15
High LDL no lipid meds	gePS	5.15	8.89E-186
High LDL no lipid meds	CarrierStatus*gePS	-15.39	0.037
High HDL	CarrierStatus	18.47	4.83E-10
High HDL	gePS	2.53	2.49E-270
High HDL	CarrierStatus*gePS	8.29	0.001
High triglycerides	CarrierStatus	135.58	6.13E-27
High triglycerides	gePS	15.34	8.76E-232
High triglycerides	CarrierStatus*gePS	34.36	0.01
Obesity	CarrierStatus	2.14	0.01
Obesity	gePS	0.78	6.74E-228
Obesity	CarrierStatus*gePS	0.61	0.50

*per SD global polygenic risk score (gePS)

Model adjusted for age, sex, PC's; Pvalues two-sided

Supplementary Table 6: Frequency cut-offs used for ClinVar variant curation.

**Phenotype (Genes - only genes with ClinVar P/LP clinical testing variants are listed)**	**GnomAD Frequency Cut-offs**			
	PM2	PM2 supporting	BS1	BA1
High LDL (*APOB*, *LDLR*)	Absent/0.0%	N/A	0.5% - 5%	>5%
Obesity (*MC4R*)	Absent/0.0%	<0.02%	N/A	N/A
High Triglycerides (*APOA5*, *LPL*)	AC<=1	N/A	N/A	N/A
MODY (*GCK*, *HNF1A*, *HNF1B*, *HNF4A*, *PDX1*; Extended: *ABCC8*, *CEL*, *INS*, *KCNJ11*)	<0.002%	N/A	0.0033% - 0.01%	>0.01%
Neonatal Diabetes (*ABCC8*, *HNF1B, INS*, *KCNJ11*)	<0.002%	N/A	0.0033% - 0.01%	>0.01%
Lipodystrophy (*LMNA*, *PPARG*)	<0.002%	N/A	0.0033% - 0.01%	>0.01%

Note: Most AMP T2D participants are included in gnomAD, so we used an adjusted gnomAD allele frequency calculated by subtracting the number of AMP T2D carriers from the number of total gnomAD carriers

Supplementary Figures

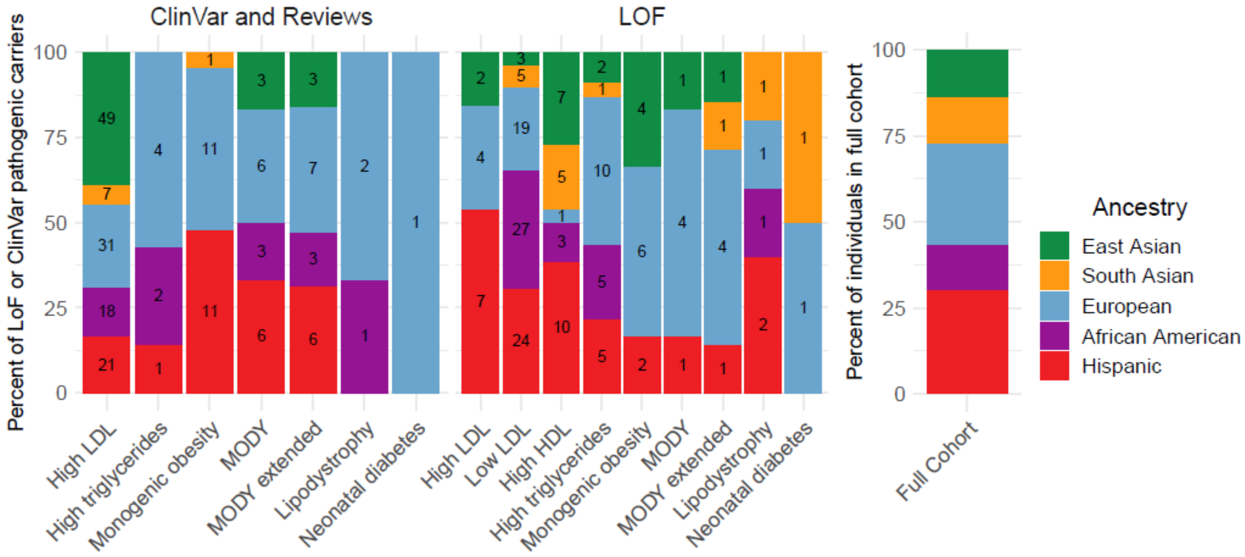

Supplementary Figure 1. Distribution of clinically significant variants across ancestries. Percent of AMP-T2D-GENES carriers in each ancestry across conditions, broken down by ClinVar/review variants (left panel) and pLoF variants (middle panel), compared to the ancestry of the full cohort (right panel).

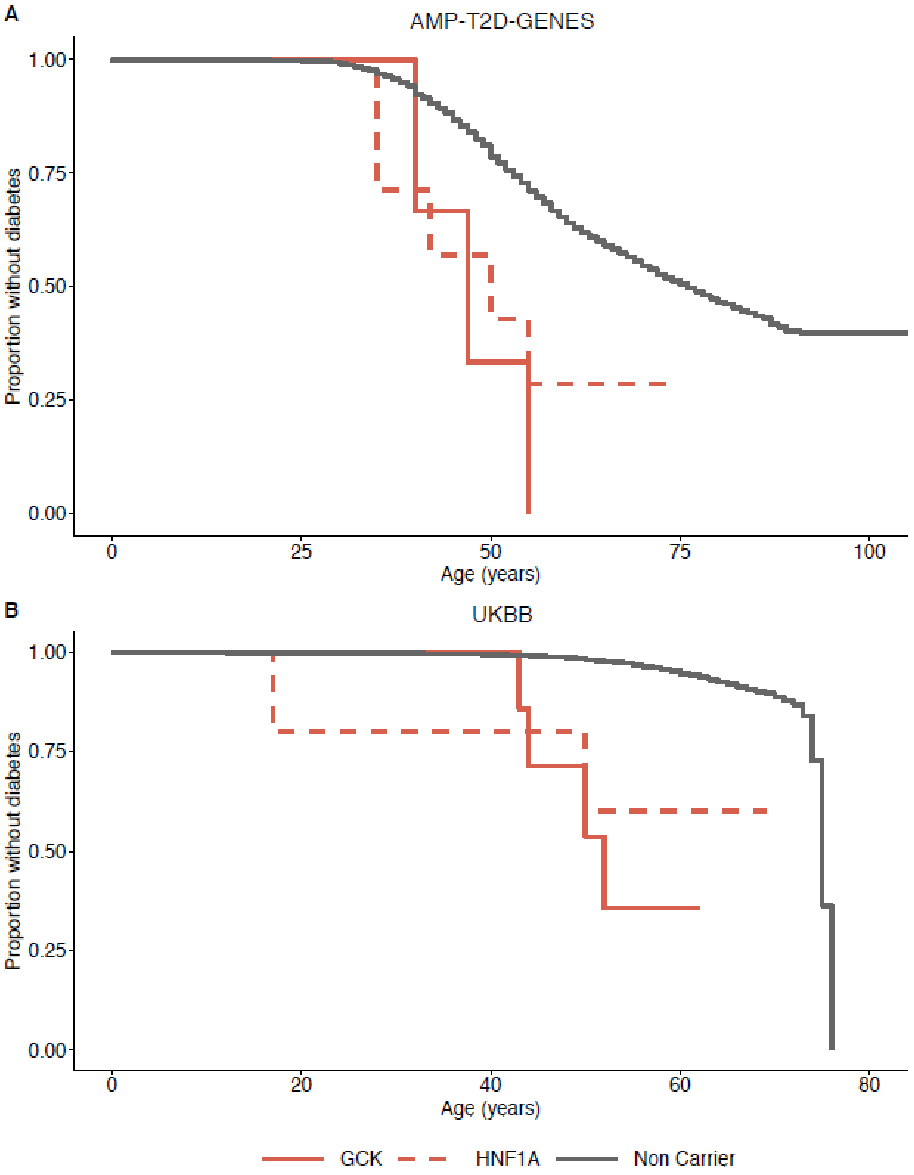

Supplementary Figure 2. Carriers of clinically significant variants in MODY genes show a younger age of diabetes diagnosis compared to the rest of the AMP-T2D-GENES cohorts and UK Biobank population.

Kaplan-Meier curves of the proportion of individuals without a diabetes diagnosis in AMP-T2D-GENES (**A**) and UK Biobank (**B**). We used age of diabetes diagnosis when reported, and for all others we used the most recent age recorded with no diabetes diagnosis. Lines are colored by clinically significant carrier status: carriers red and non-carriers grey. Line type for clinically significant carriers indicates the gene the variant falls within.

List of consortia members

AMP-T2D-GENES

Gonçalo R. Abecasis[1,2], Carlos A. Aguilar-Salinas[3], David M. Altshuler[4,5,6,7,8], Gil Atzmon[9,10,11], Francisco Barajas-Olmos[12], Aris Baras[13], Nir Barzilai[10], Graeme I. Bell[14], Thomas W. Blackwell[1], John Blangero[15,16], Michael Boehnke[17], Eric Boerwinkle[18,19], Lori L. Bonnycastle[20], Erwin P. Bottinger[21], Donald W. Bowden[22,23], Jennifer A. Brody[24], Brian Burke[25], Noël P. Burtt[7,8], David J. Carey[26], Lizz Caulkins[8], Federico Centeno-Cruz[12,27], John C. Chambers[28,29,30,31], Juliana Chan[32], Edmund Chan[33], Ling Chen[34], Siying Chen[17], Ching-Yu Cheng[35,36,37,38], Francis S. Collins[20], Cecilia Contreras-Cubas[12], Adolfo Correa[39], Maria Cortes[40], Nancy J. Cox[14,41], Emilio Córdova[12], Dana Dabelea[42,43], Paul S. de Vries[44], Ralph A. DeFronzo[45], Frederick E. Dewey[13], Lawrence Dolan[46], Kimberly L. Drews[25], Ravindranath Duggirala[15,16], Josée Dupuis[47,48,49], Ma Elena Gonzalez[50], Amanda Elliott[8,34], Maria Eugenia Garay-Sevilla[51], Jason Flannick[7,8,52,53], Jose C. Florez[4,6,7,8], James S. Floyd[54], Philippe Frossard[55], Christian Fuchsberger[1,56], Stacey B. Gabriel[40,57], Humberto García-Ortiz[12], Christian Gieger[58,59,60], Benjamin Glaser[61], Clicerio Gonzalez[62], Niels Grarup[63], Leif Groop[64,65,66], Myron Gross[67], Christopher A. Haiman[68], Sohee Han[69], Craig L. Hanis[70], Torben Hansen[63,71], Nancy L. Heard-Costa[47,49,72], Susan R. Heckbert[73], Brian E. Henderson[68], Soo Heon Kwak[74], Anne U. Jackson[75], Young Jin Kim[69,76], Marit E. Jørgensen[77,78,79], Megan Kelsey[25,42], Bong-Jo Kim[69], Ryan Koesterer[8], Heikki A. Koistinen[80,81,82], Jaspal S. Kooner[30,31,83,84], Johanna Kuusisto[85,86,87], Markku Laakso[85,86,87,88], Leslie A. Lange[89,90,91], Joseph B. Leader[26], Juyoung Lee[69], Jong-Young Lee[92,93], Donna M. Lehman[94], H. Lester Kirchner[26], Allan Linneberg[95,96,97,98], Ching-Ti Liu[48], Jianjun Liu[33,99,100], Ruth J. F. Loos[101], Valeriya Lyssenko[65,102], Ronald C. W. Ma[32], Anubha Mahajan[103], Alisa K. Manning[6,7,104,105], Juan Manuel Malacara-Hernandez[51], Anthony Marcketta[13], Angélica Martínez-Hernández[12], Karen Matsuo[17], Elizabeth Mayer-Davis[106], Mark I. McCarthy[103,107,108], James B. Meigs[6,40,109,110], Thomas Meitinger[111,112,113,114], Elvia Mendoza-Caamal[12,27], Josep M. Mercader[7,8,115,116], Hyun Min Kang[1], Karen L. Mohlke[89,117], Andrew D. Morris[118], Andrew P. Morris[119,120,121], Alanna C. Morrison[18], Anne Ndungu[121], Maggie C. Y. Ng[122], Peter Nilsson[123], Christopher J. O'Donnell[6,49,109,124,125,126,127], Colm O'Dushlaine[13], Lorena Orozco[12,27], Colin N. A. Palmer[128], James S. Pankow[129], Anthony J. Payne[121], Oluf B. Pedersen[63,130], Catherine Pihoker[131], Wendy S. Post[132], Michael Preuss[133], Bruce M. Psaty[24,134,135], Asif Rasheed[55], Alexander P. Reiner[136,137], Cristina Revilla-Monsalve[138], Stephen S. Rich[139,140], Neil R. Robertson[103], Jerome I. Rotter[141,142,143], Danish Saleheen[55,144], Nicola Santoro[145,146], Claudia Schurmann[147], Laura J. Scott[1], Mark Seielstad[148,149,150], Yoon Shin Cho[151], E. Shyong Tai[33,37,152,153], Xueling Sim[1,100], Robert Sladek[154,155,156], Kerrin S. Small[157], Xavier Soberón[12], Kyong Soo Park[84,158,159], Timothy D. Spector[157], Konstantin Strauch[59,60,160], Heather M. Stringham[1], Tim M. Strom[112,113,114], Claudia H. T. Tam[161], Tanya M. Teslovich[1,13], Farook Thameem[162], Brian Tomlinson[32], Jason M. Torres[14,121], Russell P. Tracy[163], Tiinamaija Tuomi[164,165,166], Jaakko Tuomilehto[167,168,169,170,171], Teresa Tusié-Luna[3,172], Miriam S. Udler[8,34], Rob M. van Dam[33,100,173], Ramachandran S. Vasan[49,174], Marijana Vujkovic[144], Shuai Wang[48], Ryan P. Welch[17], Jennifer Wessel[175,176], N. William Rayner[103,177], James G. Wilson[178], Daniel R. Witte[79,179,180], Tien-Yin Wong[38,153,181], Wing Yee So[161], Mi Yeong Hwang[69], Yik Ying Teo[182,183], Philip Zeitler[25,42]

T2D-GENES

Gonçalo R. Abecasis[1,2], Marcio Almeida[15], David M. Altshuler[4,5,6,7,8], Jennifer L. Asimit[184], Gil Atzmon[9,10,11], Mathew Barber[185], Nicola L. Beer[186], Graeme I. Bell[14], Jennifer Below[70], Thomas W. Blackwell[1], John Blangero[15,16], Michael Boehnke[17], Donald W. Bowden[22,23], Noël P. Burtt[7,8], John C. Chambers[28,29,30,31], Peng Chen[100], Han Chen[48], Peter S. Chines[1,20], Sungkyoung Choi[187], Claire Churchhouse[7], Pablo Cingolani[188], Belinda K. Cornes[38], Nancy J. Cox[14,41], Aaron G. Day-Williams[184], Ravindranath Duggirala[15,16], Josée Dupuis[47,48,49], Thomas Dyer[15], Shuang Feng[1], Juan Fernandez-Tajes[103], Teresa Ferreira[103], Tasha E. Fingerlin[43], Jason Flannick[7,8,52,53], Jose C. Florez[4,6,7,8], Pierre Fontanillas[7], Timothy M. Frayling[189], Christian Fuchsberger[1,56], Eric R. Gamazon[14], Kyle Gaulton[103], Saurabh Ghosh Anna Gloyn[186], Robert L.

Grossman[14], Jason Grundstad[190], Craig L. Hanis[70], Allison Heath[190], Heather Highland[70], Momoko Hirokoshi[103], Ik-Soo Huh[187], Jeroen R. Huyghe[1], Kamran Ikram[38,153,191,192], Kathleen A. Jablonski[193], Young Jin Kim[69,76], Goo Jun[1], Norihiro Kato[194], Jayoun Kim[187], Kevin Koi-Yau Lam[100], Jaspal S. Kooner[30,31,83,84], Min-Seok Kwon[187], Hae Kyung Im[195], Markku Laakso[85,86,87,88], Selyeong Lee[187], Sungyoung Lee[190], Jaehoon Lee[187], Jong-Young Lee[92,93], Donna M. Lehman[94], Heng Li[7], Cecilia M. Lindgren[103], Xuanyao Liu[196], Oren E. Livne[185], Adam E. Locke[1], Anubha Mahajan[103], Julian B. Maller[197], Alisa K. Manning[6,7,104,105], Taylor J. Maxwell[70], Alexander Mazoure[198], Mark I. McCarthy[103,107,108], James B. Meigs[6,40,109,110], Byungju Min[187], Karen L. Mohlke[89,117], Andrew P. Morris[119,120,121], Solomon K. Musani[39], Yoshihiko Nagai[198], Maggie C. Y. Ng[122], Dan Nicolae[20,185], Sohee Oh[187], Nicholette D. Palmer[199], Taesung Park[187], Toni I. Pollin[200], Inga Prokopenko[103,201], David Reich[7,202], Manuel A. Rivas[103,105,203], C. Ryan King[195], Laura J. Scott[1], Mark Seielstad[148,149,150], Yoon Shin Cho[151], E. Shyong Tai[33,37,152,153], Xueling Sim[1,100], Robert Sladek[154,155,156], Philip Smith[204], Ioanna Tachmazidou[184], Tanya M. Teslovich[1,13], Jason M. Torres[14,121], Vasily Trubetskoy[14], Sara M. Willems[205,206,207], Amy L. Williams[7,202], James G. Wilson[178], Steven Wiltshire[208], Sungho Won[209], Andrew R. Wood[189], Wang Xu[152], Yik Ying Teo[182,183], Joon Yoon[187], Matthew Zawistowski[1], Eleftheria Zeggini[184], Weihua Zhang[29], Sebastian Zöllner[210]

SIGMA

Irma Aguilar-DelfiÌĄn[27], C arlos A . A guilar-Salinas[3], D avid M . A ltshuler[4,5,6,7,8], U lices A lvirde[3], Kristin Ardlie[57], Wendy M. Brodeur[57], Noël P. Burtt[7,8], Juan Carlos Fernández-López[27], Federico Centeno-Cruz[12,27], Claire Churchhouse[7], Emilio Córdova[27], Andrew T. Crenshaw[57], Ivette Cruz-Bautista[3], MariÌĄa Elena González-Villalpando[211], Karol Estrada[6,7,212], Timothy Fennell[7], Jose C. Florez[4,6,7,8], Jennifer Franklin[57], Diane Gage[57], Humberto GarciÌĄa-OrtiÌĄz[27], Clicerio González-Villalpando[211], DonajiÌĄ Gómez[3], Christopher A. Haiman[68], Brian E. Henderson[68], Alicia Huerta-Chagoya[3,213], Sergio Islas-Andrade[138], Suzanne B. R. Jacobs[7], MariÌĄa José Gómez-Vázquez[3,214], Laurence N. Kolonel[215], Loic Le Marchand[215], Linda Liliana Muñoz-Hernández[3], MariÌĄa Luisa Ordóñez-Sánchez[3], Daniel G. MacArthur[6,7,212], Scott Mahan[57], Alisa K. Manning[6,7,104,105], Angélica MartiÌĄnez-Hernández[27], Carla Márquez- Luna[27], Elvia Mendoza-Caamal[12,27], Josep M. Mercader[7,8,115,116], Kristine Monroe[68], Hortensia Moreno-MaciÌĄas[216], Jacquelyn Murphy[7], Benjamin Neale[7,212], Robert C. Onofrio[57], Lorena Orozco[12,27], Cristina Revilla- Monsalve[138], Laura Riba[213], Stephan Ripke[7,212], Rosario RodriÌĄguez- Guillén[3], Eunice RodriÌĄguez-Arellano[217], Maribel RodriÌĄguez-Torres[3], Sandra Romero-Hidalgo[27], Tamara Sáenz[3], Xavier Soberón[27], Daniel O. Stram[68], Teresa Tusié-Luna[3,213], Lynne Wilkens[215], Amy L. Williams[7,202], Wendy Winckler[57]

GoT2D

Gonçalo R. Abecasis[1,2], Vineeta Agarwala[7], Peter Algren[64], David M. Altshuler[4,5,6,7,8], Eric Banks[57], Richard N. Bergman[68], Thomas W. Blackwell[1], Michael Boehnke[17], Lori L. Bonnycastle[20], David Buck[103], Noël P. Burtt[7,8], Peter S. Chines[1,20], Francis S. Collins[20], Mark A. DePristo[7], Peter Donnelly[103], Timothy Fennell[7], Jason Flannick[7,8,52,53], Pierre Fontanillas[7], Timothy M. Frayling[189], Christian Fuchsberger[1,56], Stacey B. Gabriel[40,57], Kyle Gaulton[103], Christian Gieger[58,59,60], Harald Grallert[59], Todd Green[7], Leif Groop[64,65,66], Christopher Hartl[7], Andrew T. Hattersley[218], Bryan Howie[185], Martin Hrabé de Angelis[59], Cornelia Huth[59], Jeroen R. Huyghe[1], Bo Isomaa[64], Anne U. Jackson[75], Goo Jun[1], Jasmina Kravic[64], Jennifer Kriebel[59], Ashish Kumar[103], Phoenix Kwan[1], Claes Ladenvall[64], Cecilia M. Lindgren[103], Adam E. Locke[1], Gerton Lunter[103], Clement Ma[1], Anubha Mahajan[103], Alisa K. Manning[6,7,104,105], Mark I. McCarthy[103,107,108], Gil McVean[103], Christa Meisinger[59], Thomas Meitinger[111,112,113,114], Hyun Min Kang[1], Karen L. Mohlke[89,117], Andrew P. Morris[119,120,121], Loukas Moutsianas[103], Martina MuÌĹller-Nurasyid[59], Pål R. Njølstad[27], Richard Pearson[103], John Perry[103], Annette Peters[59], Ryan Poplin[7], Inga Prokopenko[103,201], Wolfgang Rathmann[59], Janina Ried[59], Manuel A. Rivas[103,105,203], Neil R. Robertson[103], Laura J. Scott[1], Khalid Shakir[57], Xueling Sim[1,100], Kerrin S. Small[157], Timothy D. Spector[157], Michael Stitzel[219], Konstantin Strauch[59,60,160], Heather

M. Stringham[1], Tim M. Strom[112,113,114], Adrian Tan[1], Tanya M. Teslovich[1,13], Tiinamaija Toumi[64], Jaakko Tuomilehto[167,168,169,170,171], Martijn van de Bunt[186], N. William Rayner[103,177]

LuCAMP

Anders Albrechtsen[220,221], Gitte Andersen[130], Arne Astrup[222], Lars Bolund[223], Torben Hansen[63,71], Torben Jørgensen[98,224], Karsten Kristiansen[220], Torsten Lauritzen[225], Rasmus Nielsen[220,221], Oluf B. Pedersen[63,130], Thue W. Schwartz[226], Jun Wang[227], Daniel R. Witte[79,179,180]

ESP

BroadGO

Gonçalo R. Abecasis[1,2], Hooman Allayee[233], David M. Altshuler[4,5,6,7,8], Sharon Cresci[234], Mark J. Daly[105,203], Paul I. W. de Bakker[203,235,236], Mark A. DePristo[7], Ron Do[203], Peter Donnelly[103], Deborah N. Farlow[203], Timothy Fennell[7], Stacey B. Gabriel[40,57], Kiran Garimella[237], Stanley L. Hazen[238], Youna Hu[239], Daniel M. Jordan[235,240], Goo Jun[1], Sekar Kathiresan[105,203,235], Adam Kiezun[57], Guillaume Lettre[203,241,242], Mingyao Li[243], Bingshan Li[239], Hyun Min Kang[1], Christopher H. Newton-Cheh[105,203,235], Sandosh Padmanabhan[244,245], Gina M. Peloso[203,235,246,247,248], Sara Pulit[203], Daniel J. Rader[243], David Reich[7,202], Muredach P. Reilly[243], Manuel A. Rivas[103,105,203], Steve Schwartz[136], Laura J. Scott[1], David S. Siscovick[249,250], John A. Spertus[251], Nathaniel O. Stitziel[109], Nina Stoletzki[109,203,235], Shamil R. Sunyaev[109,203,235], Benjamin F. Voight[105,203], Cristen J. Willer[239]

HeartGO

L. Adrienne Cupples[47,48,49], Ermeg Akylbekova[252,253], Larry D. Atwood[47], Christie M. Ballantyne[254,255], Maja Barbalic[256], Emelia J. Benjamin[47], Joshua C. Bis[257], Eric Boerwinkle[18,19], Donald W. Bowden[22,23], Jennifer A. Brody[24], Matthew Budoff[258], Greg Burke[23], Sarah Buxbaum[252], Jeff Carr[23], Ida Y. Chen[141], Donna T. Chen[259], Wei-Min Chen[259], Pat Concannon[259], Jacy Crosby[256], Ralph D'Agostino[47], O. Dale Williams[260], Anita L. DeStefano[47], Albert Dreisbach[253], Josée Dupuis[47,48,49], Jaclyn Ellis[91], Aaron R. Folsom[261], Myriam Fornage[262], Ervin Fox[253], Caroline S. Fox[126], Vincent Funari[141], Santhi K. Ganesh[239], Julius Gardin[263], David Goff[23], Ora Gordon[141], R. Graham Barr[264], Wayne Grody[265], Myron Gross[67], Xiuqing Guo[141,143], Ira M. Hall[259], Nancy L. Heard-Costa[47,49,72], Susan R. Heckbert[73], Nicholas Heintz[231], David M. Herrington[23], DeMarc Hickson[252,253], Jie Huang[126], Shih-Jen Hwang[47,126], David R. Jacobs[261], Nancy S. Jenny[231], Craig W. Johnson[137], Andrew D. Johnson[126], Steven Kawut[243], Richard Kronmal[137], Raluca Kurz[141], Chrstina L Wassel[266], Leslie A. Lange[89,90,91], Ethan M. Lange[91,267], Martin G. Larson[47], Mark Lawson[259], Daniel Levy[126,268,269], Cora E. Lewis[270], Dalin Li[141], Honghuang Lin[47], Jiankang Liu[253], Kiang Liu[271], Xiaoming Liu[256], Yongmei Liu[272], Chunyu Liu[49,126,268], William T. Longstreth[137], Cay Loria[126], Thomas Lumley[273], Kathryn Lunetta[47], Rachel Mackey[274], Aaron J. Mackey[259], Ani Manichaikul[259], Taylor J. Maxwell[70], Barbara McKnight[137], James B. Meigs[6,40,109,110], Alanna C. Morrison[18], Solomon K. Musani[39], Josyf C. Mychaleckyj[259], Jennifer A. Nettleton[256], Kari North[91], Christopher J. O'Donnell[6,49,109,124,125,126,127], Daniel O'Leary[275], Frank Ong[141], Walter Palmas[276], James S. Pankow[129], Nathan D. Pankratz[277], Shom Paul[259], Marco Perez[278], Sharina D. Person[270,279], J. Peter Durda[231], Joseph Polak[275], Wendy S. Post[132],

Bruce M. Psaty[24,134,135], Aaron R. Quinlan[259], Leslie J. Raffel[141], Vasan S. Ramachandran[47], Alexander P. Reiner[136,137], Kenneth Rice[24], Stephen S. Rich[139,140], Jerome I. Rotter[141,142,143], Jill P. Sanders[231], Pamela Schreiner[261], Sudha Seshadri[47], Steve Shea[109,240], Stephen Sidney[280], Kevin Silverstein[261], David S. Siscovick[249,250], Nicholas L. Smith[137], Nona Sotoodehnia[137], Asoke Srinivasan[281], Herman A. Taylor[252,253,281], Kent D. Taylor[141,143], Fridtjof Thomas[256], Russell P. Tracy[163], Michael Y. Tsai[261], Kelly A. Volcik[256], Karol Watson[265], Gina Wei[126], Wendy White[281], Kerri L. Wiggins[231], Jemma B. Wilk[47], Gregory Wilson[252], James G. Wilson[178], Phillip Wolf[47], Neil A. Zakai[231]

ISGS and SWISS

John Hardy[282,283,284], James F. Meschia[285], Michael A. Nalls[286], Stephen S. Rich[139,140], Andrew Singleton[287], Brad Worrall[259]

LungGO

Ibrahim Abdulhamid[288], Frank Accurso[289], Ran Anbar[290], Mary Ann Passero[291], Michael J. Bamshad[131,137], Kathleen C. Barnes[292], Terri Beaty[292], Abigail Bigham[137], Phillip Black[293], Eugene Bleecker[23], Kati Buckingham[137], Daniel Caplan[294], Barbara Chatfield[295], Wei-Min Chen[259], Aaron Chidekel[296], Michael Cho[109,235], David C. Christiani[105], James D. Crapo[297], Julia Crouch[131], Denise Daley[298], Hong Dang[91], Anthony Dang[91], Alicia De Paula[299], Joan DeCelie- Germana[300], Allen Dozor[301,302], Mitch Drumm[91], Maynard Dyson[303], Julia Emerson[131,137], Mary J. Emond[137], Thomas Ferkol[234,304], Robert Fink[305], Cassandra Foster[292], Deborah Froh[259], Li Gao[292], William Gershan[306], Ronald L. Gibson[131,137], Elizabeth Godwin[91], Magdalen Gondor[307], Hector Gutierrez[270], Nadia N. Hansel[292,308], Paul M. Hassoun[292], Peter Hiatt[309], John E. Hokanson[289], Michelle Howenstine[310,311], Laura K. Hummer[292], Jamshed Kanga[312], Yoonhee Kim[313], Michael R. Knowles[91], Michael Konstan[314], Thomas Lahiri[315], Nan Laird[316], Christoph Lange[316], Xihong Lin[316], Lin Lin[235], Tin L. Louie[137], David Lynch[297], Barry Make[297], Anne Marie Cairns[317], Thomas R. Martin[137,318], Steve C. Mathai[292], Rasika A. Mathias[292,319], Sharon McNamara[131], John McNamara[320], Deborah Meyers[23], Susan Millard[321,322], Peter Mogayzel[292], Richard Moss[323], Tanda Murray[292], Dennis Nielson[150], Blakeslee Noyes[324], Wanda O'Neal[91], Brian O'Sullivan[325], David Orenstein[326], Rhonda Pace[91], Peter Pare[327], Elizabeth Perkett[328], Adrienne Prestridge[329], Nicholas M. Rafaels[292], Bonnie Ramsey[131,137], Elizabeth Regan[297], Clement Ren[330], George Retsch-Bogart[91], Michael Rock[331], Antony Rosen[292], Margaret Rosenfeld[131,137], Ingo Ruczinski[308], Andrew Sanford[298], David Schaeffer[332], Cindy Sell[91], Daniel Sheehan[333], Edwin K. Silverman[109,235], Don Sin[305], Terry Spencer[334], Jackie Stonebraker[91], Holly K. Tabor[131,137], Laurie Varlotta[335], Candelaria I. Vergara[292], Fred Wigley[292], Robert A. Wise[292], H. Worth Parker[336,337], Fred A. Wright[91], Mark M. Wurfel[137], Robert Zanni[338], Fei Zou[91]

SeattleGO

Joshua M. Akey[137], Michael J. Bamshad[131,137], Carlos D. Bustamante[278], David R. Crosslin[137], Evan E. Eichler[137], Wenqing Fu[137], Adam Gordon[137], Simon Gravel[278], Phil Green[137], Gail P. Jarvik[137], Jill M. Johnsen[137,339], Mengyuan Kan[254], Eimear E. Kenny[278], Jeffrey M. Kidd[278], Fremiet Lara-Garduno[254], Suzanne M. Leal[254], Dajiang J. Liu[254], Sean McGee[137], Deborah A. Nickerson[137], Timothy D. O'Connor[137], Bryan Paeper[137], Mark J. Rieder[340], Peggy D. Robertson[137], Jay Shendure[137], Joshua D. Smith[137], Jacob A. Tennessen[137], Emily H. Turner[137], Gao Wang[254]

WHISP

Garnet Anderson[136], Hoda Anton-Culver[341], Themistocles L. Assimes[278], Paul L. Auer[136], Shirley Beresford[136], Chris Bizon[91], Henry Black[342], Robert Brunner[343], Robert Brzyski[256], Dale Burwen[126], Bette Caan[280], Christopher S. Carlson[136,137], Cara L. Carty[136], Rowan Chlebowski[344], Steven Cummings[150], J. David

Curb[345], Charles B. Eaton[346,347], Leslie Ford[126], Nora Franceschini[91], Stephanie M. Fullerton[137], Margery Gass[348], Nancy Geller[126], Gerardo Heiss[91], Barbara V. Howard[349,350], Li Hsu[136], Carolyn M. Hutter[136], John Ioannidis[278], Rebecca Jackson[351], Shuo Jiao[136], Mary Jo O'Sullivan[352], Karen C. Johnson[353], Charles Kooperberg[136], Lewis Kuller[274], Andrea LaCroix[136], Kamakshi Lakshminarayan[261], Dorothy Lane[354], Leslie A. Lange[89,90,91], Ethan M. Lange[91,267], Norman Lasser[355], Erin LeBlanc[356], Cora E. Lewis[270], Kuo-Ping Li[91], Marian Limacher[357], Dan-Yu Lin[91], Benjamin A. Logsdon[136], Shari Ludlam[126], JoAnn E. Manson[109,316], Karen Margolis[261], Lisa Martin[358], Joan McGowan[126], Keri L. Monda[359], Jane Morley Kotchen[360], Lauren Nathan[265], Kari North[91], Judith Ockene[361,362], Ulrike Peters[136], Lawrence S. Phillips[294], Ross L. Prentice[136], Alexander P. Reiner[136,137], John Robbins[363], Jennifer G. Robinson[364], Jacques E. Rossouw[126], Haleh Sangi-Haghpeykar[254], Gloria E. Sarto[365], Sally Shumaker[23], Michael S. Simon[366], Marcia L. Stefanick[278], Evan Stein[367], Hua Tang[323], Kira C. Taylor[368], Cynthia A. Thomson[369], Timothy A. Thornton[137], Linda Van Horn[271], Mara Vitolins[23], Jean Wactawski-Wende[370], Robert Wallace[364], Sylvia Wassertheil-Smoller[47], Donglin Zeng[91]

NHLBI GO ESP Project Team

Deborah Applebaum-Bowden[126], Michael Feolo[371], Weiniu Gan[126], Dina N. Paltoo[126], Jacques E. Rossouw[126], Phyliss Sholinsky[126], Anne Sturcke[371]

Broad Genomics Platform

Adal Abebe[57], Justin Abreu[57], David An[57], Kristin Anderka[57], Scott Anderson[57], Chamara Aneesha Jayesinghe[57], Maryam Aqchour[57], Joshua Araya[57], Samuel Aronson[57], Mehrtash Babadi[57], Sarah Babchuck[57], Samira Bahl[57], Esme Baker[57], Eric Banks[57], Jessica Barbagallo[57], Alexander Baumann[57], Matthew Bemis[57], David Benjamin[57], Louis Bergelson[57], Kylee Bergin[57], Dave Bernick[57], Andrew Bernier[57], Amy Biasella[57], Jonathan Bistline[57], Brendan Blumenstiel[57], Nicole Bolliger[57], Claude Bonnet[57], Patrick Brehio[57], Wendy M. Brodeur[57], Joseph BuAbbud[57], Jody Camarata[57], Jay Carey[57], Yee-Ming Chan[57], Sheila Chandran[57], Nikita Chauhan[57], Aaron Chevalier[57], Carrie Cibulskis[57], Kristian Cibulskis[57], Michelle Cipicchio[57], Kristen Connolly[57], Maura Costello[57], Miguel Covarrubias[57], Vivek Dasari[57], Michael Dasilva[57], Tim De Smet[57], Matthew DeFelice[57], Samuel DeLuca[57], Katerina Dimitriou[57], Jacqueline Dion[57], Christine DiTondo[57], Gary Dlugy[57], Sheila Dodge[57], Teni Dowdell[57], Phil Dunlea[57], Hussein Elgridly[57], M. Erik Husby[57], Yossi Farjoun[57], Anna Farrell[57], Damien Fenske-Corbiere[57], Henry Ferrara[57], Steven Ferriera[57], Nicholas Fitzgerald[57], Mark Fleharty[57], Leo Forconesi[57], Scott Frazer[57], Stacey B. Gabriel[40,57], Laura Gauthier[57], Christina Gearin[57], Jeff Gentry[57], Linley Gerber[57], Diego Gil[57], Alexandra Gkrekos[57], Douglas Gobron[57], George Grant[57], Lisa Green[57], Liraz Greenfeld[57], Jonna Grimsby[57], Namrata Gupta[57], Kunsang Gyaltsen[57], Susanna Hamilton[57], Maegan Harden[57], Andreina Haubold[57], Soo Hee Lee[57], Jen Hendrey[57], Maria Hofbauer[57], Andrew Hollinger[57], Laurie Holmes[57], Tom Howd[57], Steve Huang[57], Dong-Keun Jang[57], Victoria Janik[57], Thibault Jeandet[57], Fontina Kelley[57], David Kennedy[57], Adam Kiezun[57], Kevinson Kim[57], David Kling[57], Jessica Klopp[57], Anna Koutoulas[57], Katie Larkin[57], Erin LaRoche[57], Katie Larsson[57], Zach Leber[57], James

Lee[57], Samuel Lee[57], Matthew Lee[57], Marcia Leffler[57], Niall Lennon[57], Frances Letendre[57], Tsamla Lhanyitsang[57], Lee Lichtenstein[57], Pei Lin[57], Christopher Llanwarne[57], Walter Lo Forte[57], Nadya Lopez Zalba[57], Sophie Low[57], Hayley Lyon[57], Jose M Soto[57], Alyssa Macbeth[57], Vasilia Magnisalis[57], Zainab Mahmod[57], Tsheko Makuwa[57], Lauren Margolin[57], Tamara Mason[57], Scott Matthews[57], Susan McDonough[57], Sheli McDonough[57], Thomas McKenna[57], Jim Meldrim[57], Atanas Mihalev[57], Mariela Mihaleva[57], Tiffany Miller[57], Tyler Miselis[57], David Mohs[57], Ruchi Munshi[57], Moran N Cabili[57], Gregory Nakashian[57], Jared Nedzel[57], Duyen Nguyen[57], Kate Noblett[57], Corey Nolet[57], Nyima Norbu[57], Sam Novod[57], Robert C. Onofrio[57], Caroline Petersen[57], Anthony Philippakis[57], Eliot Polk[57], Sam Pollock[57], Mark Puppo[57], Jason Purnell[57], Matt Putnam[57], Anabella Racioppi[57], Brian Reilly[57], David Roazen[57], Nathan Rodriguez[57], Jason Rose[57], Erika Roth[57], Valentin Ruano-Rubio[57], Gregory Rushton[57], Dennis Ryan[57], John Saccoccio[57], Ahmed Sandakli[57], Takuto Sato[57], Michael Saylor[57], Khalid Shakir[57], Megan Shand[57], Ted Sharpe[57], David Shiga[57], David Siedzik[57], Anu Singh[57], Kara Slowik[57], Andrey Smirnov[57], Sharon Stavropoulos[57], Gregory Stoneham[57], Scott Sutherland[57], Bradley Taylor[57], Joel Thibault[57], Jon Thompson[57], Kathleen Tibbetts[57], Charlotte Tolonen[57], Kristina Tracy[57], Ellen Tsai[57], Adrienne Turi[57], Geraldine Van der Auwera[57], Diolinda Vazl[57], Veronica Vicario[57], Gina Vicente[57], Andy Vo[57], Douglas Voet[57], Sarah Walker[57], Mark Walker[57,453], Cole Walsh[57], John Walsh[57], Emily Wheeler[57], Jill Whitham[57], Jane Wilkinson[57], Michael Wilson[57], David Wilson[57], Ellen Winchester[57], David Wine[57], Alicia Wong[57], Betty Woolf[57], David Zdeb[57], Andrew Zimmer[57]

Affiliations

1. Department of Biostatistics, Center for Statistical Genetics, University of Michigan, Ann Arbor, MI, USA
2. Regeneron Pharmaceuticals, Tarrytown, NY, USA
3. Instituto Nacional de Ciencias Médicas y Nutrición Salvador Zubirán, Mexico City, Mexico
4. Center for Human Genetic Research, Massachusetts General Hospital, Boston, MA, USA
5. Department of Biology, Massachusetts Institute of Technology, Cambridge, MA, USA
6. Department of Medicine, Harvard Medical School, Boston, MA, USA
7. Program in Medical and Population Genetics, Broad Institute of Harvard and MIT, Cambridge, MA, USA
8. Programs in Metabolism and Medical and Population Genetics, Broad Institute of MIT and Harvard, Cambridge, MA, USA
9. Department of Medicine, Department of Genetics, Albert Einstein College of Medicine, New York, NY, USA
10. Departments of Medicine and Genetics, Albert Einstein College of Medicine, NY, USA
11. University of Haifa, Faculty of Natural Science, Haifa, Isarel
12. Instituto Nacional de Medicina Genómica, Mexico City, Mexico
13. Regeneron Genetics Center, Regeneron Pharmaceuticals, Tarrytown, NY, USA
14. Department of Medicine, University of Chicago, Chicago, IL, USA
15. Department of Genetics, Texas Biomedical Research Institute, San Antonio, TX, USA

16. Department of Human Genetics and South Texas Diabetes and Obesity Institute, University of Texas Rio Grande Valley, Edinburg and Brownsville, TX, USA

17. Department of Biostatistics and Center for Statistical Genetics, University of Michigan, Ann Arbor, MI, USA

18. Human Genetics Center, School of Public Health, University of Texas Health Science Center, San Antonio, TX, USA

19. Human Genome Sequencing Center, Baylor College of Medicine, Houston, TX, USA

20. National Human Genome Research Institute, National Institutes of Health, Bethesda, MD, USA

21. The Charles Bronfman Institute for Personalized Medicine, Icahn School of Medicine at Mount Sinai, New York, NY, USA

22. Department of Biochemistry, Wake Forest School of Medicine, Winston-Salem, NC, USA

23. Wake Forest University, Winston-Salem, NC, USA

24. Cardiovascular Health Research Unit, University of Washington, Seattle, WA, USA

25. Biostatistics Center, George Washington University, Washington, DC , USA

26. Geisinger Health System, Danville, PA, USA

27. Instituto Nacional de Medicina Genómica, Mexico City, Mexico

28. Department of Cardiology, Ealing Hospital NHS Trust, Southall, Middlesex, UK

29. Department of Epidemiology and Biostatistics, Imperial College London, London, UK

30. Ealing Hospital National Health Service (NHS) Trust, Middlesex, UK

31. Imperial College Healthcare NHS Trust, London, UK

32. Department of Medicine and Therapeutics, Chinese University of Hong Kong, Hong Kong, China

33. Department of Medicine, Yong Loo Lin School of Medicine, National University of Singapore, National University Health System, Singapore

34. Diabetes Research Center (Diabetes Unit), Department of Medicine, Massachusetts General Hospital, Boston, MA, USA

35. Office of Clinical Sciences, Duke National University of Singapore Graduate Medical School, National University of Singapore, Singapore

36. Ophthalmology and Visual Sciences Academic Clinical Program (Eye ACP), Duke National University of Singapore Graduate Medical School, Singapore

37. Saw Swee Hock School of Public Health, National University of Singapore and National University Health System, Singapore

38. Singapore Eye Research Institute, Singapore National Eye Centre, Singapore

39. Department of Medicine, University of Mississippi Medical Center, Jackson, MS, USA

40. Broad Institute of MIT and Harvard, Cambridge, MA, USA

41. Vanderbilt Genetics Institute, Vanderbilt University, Nashville, TN, USA
42. Children's Hospital Colorado, Aurora, CO, USA
43. Department of Epidemiology, Colorado School of Public Health, Aurora, CO, USA
44. Human Genetics Center, Department of Epidemiology Human Genetics and Environmental Sciences, School of Public Health, University of Texas Health Science Center, San Antonio, TX, USA
45. Department of Medicine, University of Texas Health Science Center, San Antonio, TX, USA
46. Cincinnati Children's Hospital Medical Center, Cincinnati, OH, USA
47. Boston University, Boston, MA, USA
48. Department of Biostatistics, Boston University School of Public Health, Boston, MA, USA
49. National Heart, Lung, and Blood Institute Framingham Heart Study, Framingham, MA, USA
50. Centro de Estudios en Diabetes, Mexico City, Mexico
51. Departments of Medicine and Human Genetics, University of Chicago, Chicago, IL, USA
52. Department of Pediatrics, Harvard Medical School, Boston, MA, USA
53. Division of Genetics and Genomics, Boston Children's Hospital, Boston, MA, USA
54. Department of Medicine and Epidemiology, University of Washington, Seattle, WA, USA
55. Center for Non-Communicable Diseases, Karachi, Pakistan
56. Institute for Biomedicine, Eurac Research, Bolzano, Italy
57. Genomics Platform, Broad Institute of Harvard and MIT, Cambridge, MA, USA
58. German Center for Diabetes Research (DZD e.V.), Neuherberg, Germany
59. Institute of Genetic Epidemiology, Helmholtz Zentrum MuÌĹnchen, Neuherberg, Germany
60. Research Unit of Molecular Epidemiology, Institute of Epidemiology, Helmholtz Zentrum München, German Research Center for Environmental Health, Neuherberg, Germany
61. Endocrinology and Metabolism Service, Hadassah-Hebrew University Medical Center, Jerusalem, Israel
62. Unidad de Diabetes y Riesgo Cardiovascular, Instituto Nacional de Salud Pública, Cuernavaca, Morelos, Mexico
63. The Novo Nordisk Foundation Center for Basic Metabolic Research, Faculty of Health and Medical Sciences, University of Copenhagen, Copenhagen, Denmark
64. Department of Clinical Sciences, Diabetes and Endocrinology, Clinical Research Centre, Lund University, Malmö, Sweden
65. Department of Clinical Sciences, Diabetes and Endocrinology, Lund University Diabetes Centre, Malmö, Sweden
66. Institute for Molecular Genetics Finland, University of Helsinki, Helsinki, Finland

67. Department of Laboratory Medicine and Pathology, University of Minnesota, Minneapolis, MN, USA

68. Department of Preventive Medicine, Keck School of Medicine, University of Southern California, Los Angeles, CA, USA

69. Division of Genome Research, Center for Genome Science, National Institute of Health, Chungcheongbuk-do, Republic of Korea

70. Human Genetics Center, University of Texas Health Science Center, San Antonio, TX, USA

71. Faculty of Health Sciences, University of Southern Denmark, Odense, Denmark

72. Department of Neurology, Boston University School of Medicine, Boston, MA, USA

73. Cardiovascular Health Research Unit and Department of Epidemiology, University of Washington, Seattle, WA, USA

74. Department of Internal Medicine, Seoul National University Hospital, Seoul, Republic of Korea

75. Department of Pathology, University of Michigan, Ann Arbor, MI, USA

76. Department of Neurology, Konkuk University School of Medicine, Seoul, South Korea

77. Greenland Centre for Health Research, University of Greenland, Nuuk, Greenland

78. National Institute of Public Health, University of Southern Denmark, Odense, Denmark

79. Steno Diabetes Center, Gentofte, Denmark

80. Department of Public Health Solutions, National Institute for Health and Welfare, Helsinki, Finland

81. Minerva Foundation Institute for Medical Research, Helsinki, Finland

82. University of Helsinki and Department of Medicine, Helsinki University Central Hospital, Helsinki, Finland

83. National Heart and Lung Institute (NHLI), Imperial College London, Hammersmith Hospital, London, UK

84. National Heart and Lung Institute, Cardiovascular Sciences, Hammersmith Campus, Imperial College London, London, UK

85. Department of Medicin, Kuopio University Hospital, Kuopio, Finland

86. Institute of Clinical Medicine, Internal Medicine, University of Eastern Finland and Kuopio University Hospital, Kuopio, Finland

87. Institute of Clinical Medicine, Internal Medicine, University of Eastern Finland, Kuopio, Finland

88. Department of Medicine, University of Eastern Finland, Kuopio Campus and Kuopio University Hospital, Kuopio, Finland

89. Department of Genetics, University of North Carolina, Chapel Hill, NC, USA

90. Department of Medicine, University of Colorado Denver, Anschutz Medical Campus, Aurora, CO, USA

91. University of North Carolina Chapel Hill, Chapel Hill, NC, USA

92. Center for Genome Science, Korea National Institute of Health, Osong Health Technology Administration Complex, Chungcheongbuk-do, South Korea

93. Department of Business Data Convergence, Chungbuk National University, Gyeonggi-do, Republic of Korea

94. Division of Clinical Epidemiology, Department of Medicine, University of Texas Health Science Center, San Antonio, TX, USA

95. Center for Clinical Research and Prevention, Bispebjerg and Frederiksberg Hospital, Copenhagen, Denmark

96. Department of Clinical Experimental Research, Rigshospitalet, Copenhagen, Denmark

97. Department of Clinical Medicine, Faculty of Health and Medical Sciences, University of Copenhagen, Copenhagen, Denmark

98. Research Centre for Prevention and Health, Glostrup University Hospital, Glostrup, Denmark

99. Genome Institute of Singapore, Agency for Science Technology and Research, Singapore

100. Saw Swee Hock School of Public Health, National University of Singapore, Singapore

101. The Mindich Child Health and Development Institute, Icahn School of Medicine at Mount Sinai, New York, NY, USA

102. University of Bergen, Bergen, Norway

103. Wellcome Trust Centre for Human Genetics, University of Oxford, Oxford, UK

104. Clinical and Translational Epidemiology Unit, Massachusetts General Hospital, Harvard University, Cambridge, MA, USA

105. Massachusetts General Hospital, Boston, MA, USA

106. University of North Carolina, Chapel Hill, NC, USA

107. Oxford NIHR Biomedical Research Centre, Churchill Hospital, Headington, UK

108. Oxford NIHR Biomedical Research Centre, Oxford University Hospitals Trust, Oxford, UK

109. Brigham and Women's Hospital, Boston, MA, USA

110. General Medicine Division, Massachusetts General Hospital, Boston, MA, USA

111. Deutsches Forschungszentrum für Herz-Kreislauferkrankungen (DZHK), Partner Site Munich Heart Alliance, Munich, Germany

112. Human Genetics, Helmholtz Zentrum MuÌĹnchen, Neuherberg, Germany

113. Institute of Human Genetics, Helmholtz Zentrum München, German Research Center for Environmental Health, Neuherberg, Germany

114. Institute of Human Genetics, Technische Universität München, Munich, Germany

115. Center for Human Genetic Research and Diabetes Research Center (Diabetes Unit), Massachusetts General Hospital, Boston, MA, USA

116. Joint BSC-CRG-IRB Research Program in Computational Biology, Barcelona Supercomputing Center, Barcelona, Spain

117. Department of Genetics, University of North Carolina Chapel Hill, Chapel Hill, NC, USA

118. Clinical Research Centre, Centre for Molecular Medicine, Ninewells Hospital and Medical School, Dundee, UK

119. Department of Biostatistics, University of Liverpool, Liverpool, UK

120. Department of Genetic Medicine, Manchester Academic Health Sciences Centre, Manchester, UK

121. Wellcome Centre for Human Genetics, Nuffield Department of Medicine, University of Oxford, Oxford, UK

122. Center for Genomics and Personalized Medicine Research, Center for Diabetes Research, Department of Biochemistry, Department of Internal Medicine, Wake Forest School of Medicine, Winston-Salem, NC, USA

123. Department of Clinical Sciences, Medicine, Lund University, Malmö, Sweden

124. Cardiology Division, Massachusetts General Hospital, Boston, MA, USA

125. Intramural Administration Management Branch, National Heart, Lung, and Blood Institute, NIH, Framingham, MA, USA

126. National Heart, Lung, and Blood Institute, Bethesda, MD, USA

127. Section of Cardiology, Department of Medicine, VA Boston Healthcare, Boston, MA, USA

128. Pat Macpherson Centre for Pharmacogenetics and Pharmacogenomics, Medical Research Institute, Ninewells Hospital and Medical School, Dundee, UK

129. Division of Epidemiology and Community Health, School of Public Health, University of Minnesota, Minneapolis, MN, USA

130. Hagedorn Research Institute, Gentofte, Denmark

131. Seattle Children's Hospital, Seattle, WA, USA

132. Division of Cardiology, Department of Medicine, Johns Hopkins University, Baltimore, MD, USA

133. Charles R. Bronfman Institute of Personalized Medicine, Icahn School of Medicine at Mount Sinai, New York, NY, USA

134. Group Health Research Institute, Seattle, WA, USA

135. Kaiser Permanente Washington Health Research Institute, Seattle, WA, USA

136. Fred Hutchinson Cancer Research Center, Seattle, WA, USA

137. University of Washington, Seattle, WA, USA

138. Instituto Mexicano del Seguro Social SXXI, Mexico City, Mexico

139. Center for Public Health Genomics, Department of Public Health Sciences, University of Virginia, Charlottesville, VA, USA

140. Center for Public Health Genomics, University of Virginia School of Medicine, Charlottesville, VA, USA

141. Cedars-Sinai Medical Center, Los Angeles, CA, USA

142. Departments of Pediatrics and Medicine, Institute for Translational Genomics and Population Sciences, Los Angeles BioMedical Research Institute at Harbor-UCLA Medical Center, Torrance, CA, USA

143. Institute for Translational Genomics and Population Sciences, Los Angeles Biomedical Research Institute at Harbor-UCLA Medical Center, Torrance, CA, USA

144. Department of Biostatistics and Epidemiology, University of Pennsylvania, Philadelphia, PA, USA

145. Department of Pediatrics, Yale University, New Haven, CT, USA

146. Yale School of Medicine, New Haven, CT, USA

147. The Genetics of Obesity and Related Metabolic Traits Program, Icahn School of Medicine at Mount Sinai, New York, NY, USA

148. Blood Systems Research Institute, San Francisco, CA, USA

149. Department of Laboratory Medicine and Institute for Human Genetics, University of California, San Francisco, San Francisco, CA, USA

150. University of California San Francisco, San Francisco, CA, USA

151. Department of Biomedical Science, Hallym University, Gangwon-do, South Korea

152. Department of Medicine, Yong Loo Lin School of Medicine, National University of Singapore, Singapore

153. Duke National University of Singapore Graduate Medical School, Singapore

154. Department of Human Genetics, McGill University, Montréal, Québec, Canada

155. Department of Medicine, Royal Victoria Hospital, Montréal, Québec, Canada

156. McGill University and Génome Québec Innovation Centre, Montreal, Quebec, Canada

157. Department of Twin Research and Genetic Epidemiology, King's College London, London, UK

158. Department of Internal Medicine, Seoul National University College of Medicine, Seoul, Republic of Korea

159. Department of Molecular Medicine and Biopharmaceutical Sciences, Graduate School of Convergence Science and Technology, Seoul National University, Seoul, South Korea

160. Institute of Medical Informatics, Biometry and Epidemiology, Chair of Genetic Epidemiology, Ludwig-Maximilians-Universität, Neuherberg, Germany

161. Hong Kong Institute of Diabetes and Obesity, Chinese University of Hong Kong, Hong Kong, China

162. Department of Biochemistry, Faculty of Medicine, Health Science Center, Kuwait University, Safat, Kuwait

163. Department of Pathology and Laboratory Medicine, Robert Larner, M.D. College of Medicine, University of Vermont, Burlington, VT, USA

164. Department of Endocrinology, Abdominal Centre, Helsinki University Hospital, Helsinki, Finland

165. Folkhälsan Research Centre, Helsinki, Finland

166. Research Programs Unit, Diabetes and Obesity, University of Helsinki, Helsinki, Finland

167. Center for Vascular Prevention, Danube University Krems, Krems, Austria

168. Department of Public Health, University of Helsinki, Helsinki, Finland

169. Diabetes Prevention Unit, National Institute for Health and Welfare, Helsinki, Finland

170. Diabetes Research Group, King Abdulaziz University, Jeddah, Saudi Arabia

171. Instituto de Investigacion Sanitaria del Hospital Universario LaPaz (IdiPAZ), University Hospital La-Paz, Autonomous University of Madrid, Madrid, Spain

172. Instituto de Investigaciones Biomédicas, Departamento de Medicina Genómica y ToxicologiÌĄa, Universidad Nacional Autónoma de México, Mexico City, Mexico

173. Department of Nutrition, Harvard School of Public Health, Boston, MA, USA

174. Preventive Medicine and Epidemiology, Medicine, Boston University School of Medicine, Boston, MA, USA

175. Department of Epidemiology, Fairbanks School of Public Health, Indiana University, Indianapolis, IN, USA

176. Department of Medicine, Indiana University School of Medicine, Indianapolis, IN, USA

177. Department of Human Genetics, Wellcome Trust Sanger Institute, Hinxton, Cambridgeshire, UK

178. Department of Physiology and Biophysics, University of Mississippi Medical Center, Jackson, MS, USA

179. Danish Diabetes Academy, Odense, Denmark

180. Department of Public Health, Aarhus University, Aarhus, Denmark

181. Department of Ophthalmology, Yong Loo Lin School of Medicine, National University of Singapore, National University Health System, Singapore

182. Department of Statistics and Applied Probability, National University of Singapore, Singapore

183. Genome Institute of Singapore, Agency for Science, Technology and Research, Singapore

184. Wellcome Trust Sanger Institute, Hinxton, UK

185. Department of Human Genetics, University of Chicago, Chicago, IL, USA

186. Oxford Centre for Diabetes, Endocrinology and Metabolism, University of Oxford, Oxford, UK

187. Seoul National University, Seoul, South Korea

188. McGill Centre for Bioinformatics, McGill University, Montréal, Québec, Canada

189. Genetics of Complex Traits, University of Exeter Medical School, University of Exeter, Exeter, UK

190. Institute for Genomics and Systems Biology, University of Chicago, Chicago, IL, USA

191. Department of Ophthalmology, Erasmus Medical Center, Rotterdam, Netherlands

192. Department of Ophthalmology, National University of Singapore and National University Health System, Singapore

193. The Biostatistics Center, George Washington University, Washington, DC , USA

194. Department of Gene Diagnostics and Therapeutics, Research Institute, National Center for Global Health and Medicine, Tokyo, Japan

195. Department of Health Studies, University of Chicago, Chicago, IL, USA

196. Graduate School for Integrative Science and Engineering, National University of Singapore, Singapore

197. Department of Statistics, University of Oxford, Oxford, UK

198. McGill University, Montréal, Québec, Canada

199. Center for Genomics and Personalized Medicine Research, Wake Forest School of Medicine, Winston-Salem, NC, USA

200. Department of Medicine, Program for Personalized and Genomic Medicine, University of Maryland School of Medicine, Baltimore, MD, USA

201. Department of Medical Sciences, Molecular Epidemiology and Science for Life Laboratory, Uppsala University, Uppsala, Sweden

202. Department of Genetics, Harvard Medical School, Boston, MA, USA

203. Broad Institute of Harvard and MIT, Cambridge, MA, USA

204. National Institute of Diabetes and Digestive and Kidney Disease, National Institutes of Health, Bethesda, MD, USA

205. Department of Genetic Epidemiology, Erasmus Medical Center, Rotterdam, Netherlands

206. Genetic Epidemiology Unit, Department of Epidemiology, Erasmus University Medical Center, Rotterdam, The Netherlands

207. MRC Epidemiology Unit, University of Cambridge School of Clinical Medicine, Institute of Metabolic Science, Cambridge Biomedical Campus, Cambridge, UK

208. Centre for Medical Research, Western Australian Institute for Medical Research, University of Western Australia, Nedlands, Australia

209. Chung-Ang University, Seoul, South Korea

210. Department of Psychiatry, University of Michigan, Ann Arbor, MI, USA

211. Centro de Estudios en Diabetes, Unidad de Investigacion en Diabetes y Riesgo Cardiovascular, Centro de Investigacion en Salud Poblacional, Instituto Nacional de Salud Publica, Mexico City, Mexico

212. Analytic and Translational Genetics Unit, Massachusetts General Hospital, Boston, MA, USA

213. Instituto de Investigaciones Biomédicas, Unidad de BiologiÌĄa Molecular y Medicina Genómica, UNAM/INCMNSZ, Mexico City, Mexico

214. Universidad Autónoma de Nuevo León, San Nicolás de los Garza, Nuevo León, México

215. Epidemiology Program, University of Hawaii Cancer Center, Honolulu, HI, USA

216. Universidad Autonoma Metropolitana, Mexico City, Mexico

217. Instituto de Seguridad y Servicios Sociales para los Trabajadores del Estado, Mexico City, Mexico

218. Genetics of Diabetes, University of Exeter Medical School, University of Exeter, Exeter, UK

219. Department of Biochemistry and Molecular Biology, Pennsylvania State University, State College, PA, USA

220. Department of Biology, University of Copenhagen, Copenhagen, Denmark

221. Department of Statistics, University of California Berkeley, Berkeley, CA, USA

222. Department of Human Nutrition, Faculty of Life Sciences, University of Copenhagen, Copenhagen, Denmark

223. Department of Human Genetics, University of Aarhus, Aarhus, Denmark

224. Faculty of Medicine, University of Aalborg, Aalborg, Denmark

225. Faculty of Health Sciences, University of Aarhus, Aarhus, Denmark

226. Marie Krogh Center for Metabolic Research, Metabolic Receptology and Enteroendocrinology, Faculty of Health Sciences, University of Copenhagen, Copenhagen, Denmark

227. BGI-Shenzhen, Shenzhen, China

228. Kaiser Permanente Southern California, Pasadena, CA, USA

229. Wake Forest School of Medicine, Winston-Salem, NC, USA

230. Children's Hospital of Philadelphia, Philadelphia, PA, USA

231. University of Vermont, Burlington, VT, USA

232. University of Maryland School of Medicine, Baltimore, MD, USA

233. University of Southern California, Los Angeles, CA, USA

234. Washington University School of Medicine, St. Louis, MO, USA

235. Harvard Medical School, Boston, MA, USA

236. University Medical Center Utrecht, Utrecht, Netherlands

237. University of Oxford, Oxford, UK

238. Cleveland Clinic, Cleveland, OH, USA

239. University of Michigan, Ann Arbor, MI, USA

240. Harvard University, Cambridge, MA, USA
241. Montreal Heart Institute, Montréal, QC, Canada
242. Université de Montréal, Montreal, QC, Canada
243. University of Pennsylvania, Philadelphia, PA, USA
244. Institute of Cardiovascular and Medical Sciences, University of Glasgow, Glasgow, UK
245. University of Glasgow School of Medicine, Glasgow, UK
246. Department of Internal Medicine, Erasmus University Medical Center, Rotterdam, The Netherlands
247. Diabetes Unit, Department of Medicine, Massachusetts General Hospital, Boston, MA, USA
248. Research Unit, Skellefteå, Sweden
249. Cardiovascular Health Research Unit, Departments of Medicine and Epidemiology, University of Washington, Seattle, WA, USA
250. New York Academy of Medicine, New York, NY, USA
251. University of Missouri Kansas City, Kansas City, MO, USA
252. Jackson State University, Jackson, MS, USA
253. University of Mississippi Medical Center, Jackson, MS, USA
254. Baylor College of Medicine, Houston, TX, USA
255. Methodist DeBakey Heart Center, Houston, TX, USA
256. University of Texas Health Science Center, San Antonio, TX, USA
257. Department of Medicine, University of Washington, Seattle, WA, USA
258. Harbor-UCLA Medical Center, Torrance, CA, USA
259. University of Virginia, Charlottesville, VA, USA
260. Florida International University, Miami, FL, USA
261. University of Minnesota, Minneapolis, MN, USA
262. Brown Foundation Institute of Molecular Medicine, University of Texas Health Science Center, San Antonio, TX, USA
263. Hackensack University Medical Center, Hackensack, NJ, USA
264. Columbia University Medical Center, New York, NY, USA
265. University of California Los Angeles, Los Angeles, CA, USA
266. University of California San Diego, La Jolla, CA, USA
267. Department of Genetics and Department of Biostatistics, University of North Carolina, Chapel Hill, NC, USA
268. Framingham Heart Study, Framingham, MA, USA

269. Population Sciences Branch, National Heart, Lung, and Blood Institute, National Institutes of Health, Bethesda, MD, USA

270. University of Alabama at Birmingham, Birmingham, AL, USA

271. Northwestern University, Evanston, IL, USA

272. Department of Epidemiology and Prevention, Division of Public Health Sciences, Wake Forest University, Winston-Salem, NC, USA

273. University of Auckland, Aukland, New Zealand

274. University of Pittsburgh, Pittsburgh, PA, USA

275. Tufts University School of Medicine, Boston, MA, USA

276. Columbia University, New York, NY, USA

277. Indiana University School of Medicine, Indianapolis, IN, USA

278. Stanford University School of Medicine, Stanford, CA, USA

279. University of Alabama at Tuscaloosa, Tuscaloosa, AL, USA

280. Kaiser Permanente Division of Research, Oakland, CA, USA

281. Tougaloo College, Tougaloo, MS, USA

282. Institute of Neurology, London, UK

283. Reta Lila Weston Research Laboratories, London, UK

284. University College London, London, UK

285. Mayo Clinic, Rochester, MN, USA

286. Laboratory of Neurogenetics, National Institute on Aging, Bethesda, MD, USA

287. National Institute on Aging, Bethesda, MD, USA

288. Children's Hospital of Michigan, Detroit, MI, USA

289. University of Colorado, Boulder, CO, USA

290. Upstate Medical University, Oneida, NY, USA

291. Rhode Island Hospital, Providence, RI, USA

292. Johns Hopkins University, Baltimore, MD, USA

293. Children's Mercy Hospital, Kansas City, MO, USA

294. Emory University, Atlanta, GA, USA

295. University of Utah, Salt Lake City, UT, USA

296. A.I. Dupont Institute Medical Center, Wilmington, DE, USA

297. National Jewish Health, Denver, CO, USA

298. University of British Columbia, Vancouver, BC, Canada

299. Ochsner Health System, Jefferson Parish, LA, USA

300. Schneider Children's Hospital, Queens, NY, USA

301. New York Medical College, Valhalla, NY, USA

302. Westchester Medical Center, Valhalla, NY, USA

303. Cook Children's Med. Center, Fort Worth, TX, USA

304. St. Louis Children's Hospital, St. Louis, MO, USA

305. Children's Medical Center of Dayton, Dayton, OH, USA

306. Children's Hospital of Wisconsin, Milwaukee, WI, USA

307. All Children's Hospital Cystic Fibrosis Center, St Petersburg, FL, USA

308. Johns Hopkins University School of Public Health, Baltimore, MD, USA

309. Texas Children's Hospital, Houston, TX, USA

310. Indiana University, Indianapolis, IN, USA

311. Riley Hospital for Children, Indianapolis, IN, USA

312. University of Kentucky, Lexington, KY, USA

313. National Human Genome Research Institute, Bethesda, MD, USA

314. Rainbow Babies and Children's Hospital, Cleveland, OH, USA

315. Vermont Children's Hospital at Fletcher Allen Health Care, VT, USA

316. Harvard School of Public Health, Boston, MA, USA

317. Maine Medical Center, Portland, ME, USA

318. VA Puget Sound Medical Center, Seattle, WA, USA

319. The GeneSTAR Research Program, Division of General Internal Medicine, Department of Medicine, Johns Hopkins University School of Medicine, Baltimore, MD, USA

320. Children's Hospitals and Clinics of Minnesota, Minneapolis, MN, USA

321. DeVos Children's Butterworth Hospital, Grand Rapids, MI, USA

322. Spectrum Health Systems, Worcester, MA, USA

323. Stanford University, Stanford, CA, USA

324. Cardinal Glennon Children's Hospital, St. Louis, MO, USA

325. University of Massachusetts Memorial Health Care, MA, USA

326. Children's Hospital of Pittsburgh, Pittsburgh, PA, USA

327. St. Paul's Hospital, Vancouver, BC, Canada

328. Vanderbilt University, Nashville, TN, USA
329. Children's Memorial Hospital, Chicago, IL, USA
330. University of Rochester, Rochester, NY, USA
331. University of Wisconsin Hospital and Clinics, Madison, WI, USA
332. Nemours Children's Clinic, Jacksonville, FL, USA
333. Children's Hospital of Buffalo, Buffalo, NY, USA
334. Elliot Health System, Manchester, NH, USA
335. St. Christopher's Hospital for Children, Philadelphia, PA, USA
336. Dartmouth-Hitchcock Medical Center, Lebanon, NH, USA
337. New Hampshire Cystic Fibrosis Center, Nashua, NH, USA
338. Monmouth Medical Center, Long Branch, NJ, USA
339. Puget Sound Blood Center, Seattle, WA, USA
340. Adaptive Biotechnologies Corporation, Seattle, WA, USA
341. University of California Irvine, Irvine, CA, USA
342. Rush Medical Center, Chicago, IL, USA
343. University of Nevada, Reno, NV, USA
344. Los Angeles Biomedical Research Institute, Los Angeles, CA, USA
345. University of Hawaii, Honolulu, HI, USA
346. Brown University, Providence, RI, USA
347. Memorial Hospital of Rhode Island, Pawtucket, RI, USA
348. University of Cincinnati, Cincinnati, OH, USA
349. Howard University, Washington, DC, USA
350. MedStar Research Institute, Hyattsville, MD, USA
351. Ohio State University, Columbus, OH, USA
352. University of Miami, Coral Gables, FL, USA
353. University of Tennessee Health Science Center, Memphis, TN, USA
354. State University of New York at Stony Brook, Stony Brook, NY, USA
355. University of Medicine and Dentistry of New Jersey, Newark, NJ, USA
356. Kaiser Permanente Center for Health Research, Portland, OR, USA
357. University of Florida, Gainesville, FL, USA

358. George Washington University Medical Center, Washington, DC, USA

359. Amgen Inc., Newbury Park, CA, USA

360. Medical College of Wisconsin, Wauwatosa, WI, USA

361. Fallon Clinic, Worcester, MA, USA

362. University of Massachusetts, Amherst, MA, USA

363. University of California Davis, Davis, CA, USA

364. University of Iowa, Iowa City, IA, USA

365. University of Wisconsin, Madison, WI, USA

366. Wayne State University, Detroit, MI, USA

367. Medpace Reference Laboratories, Cincinnati, OH, USA

368. University of Louisville, Louisville, KY, USA

369. University of Arizona, Tucson, AZ, USA

370. University of Buffalo, Buffalo, NY, USA

371. National Center for Biotechnology Information, Bethesda, MD, USA

372. Department of Medicine and Department of Biomedical Sciences, Cedars-Sinai Medical Center, Los Angeles, CA, USA

373. Materials and Process Simulation Center, California Institute of Technology, Pasadena, CA, USA

374. Division of Statistical Genomics and Department of Genetics, Washington University School of Medicine, St. Louis, MO, USA

375. Genetics, PCPS, GlaxoSmithKline, RTP, NC, USA

376. Department of Preventive Medicine, Northwestern University Feinberg School of Medicine, Chicago, IL, USA

377. Division of Genetics and Cell Biology, San Raffaele Research institute, Milano, Italy

378. CEA, Institut de Génomique, Centre National de Génotypage, Cedex, France

379. McKusick-Nathans Institute of Genetic Medicine, Johns Hopkins University School of Medicine, Baltimore, MD, USA

380. Predoctoral Training Program in Human Genetics, McKusick-Nathans Institute of Genetic Medicine, Johns Hopkins University School of Medicine, MD, USA

381. Department of Epidemiology, German Institute of Human Nutrition Potsdam RehbruÌĹcke, Nuthetal, Germany

382. Section of Biology and Genetics, Department of Life and Reproduction Sciences, University of Verona, Verona, Italy

383. Department of Genome Dynamics, Lawrence Berkeley National Laboratory, Berkeley, CA, USA

384. Division of Preventive Medicine, Brigham and Women's Hospital, Boston, MA, USA

385. Harokopio University, Athens, Greece

386. Department of Epidemiology, Erasmus University Medical Center, Rotterdam, The Netherlands

387. Princess Al-Jawhara Al-Brahim Centre of Excellence in Research of Hereditary Disorders (PACER-HD), King Abdulaziz University, Jeddah, Saudi Arabia

388. William Harvey Research Institute, Barts and The London School of Medicine and Dentistry, Queen Mary University of London, UK

389. Statistical Genetics, PCPS, GlaxoSmithKline, RTP, NC, USA

390. Division of Cardiology, Geneva University Hospital, Geneva, Switzerland

391. McKusick-Nathans Institute of Genetic Medicine, Johns Hopkins University, Baltimore, MD, USA

392. Icelandic Heart Association, 201 Kopavogur, Iceland

393. Department of Clinical Sciences, Genetic and Molecular Epidemiology Unit, Skåne University Hospital, Malmö, Sweden

394. Department of Nutrition and Dietetics, School of Health Science and Education, Harokopio University, Athens, Greece

395. Department of Clinical and Experimental Medicine, University of Pisa School of Medicine, Pisa, Italy

396. Department of Genome Sciences, University of Washington, Seattle, WA, USA

397. Department of Public Health and Clinical Medicine, Umeå University, Umeå, Sweden

398. Atherosclerosis Research Unit, Department of Medicine Solna, Karolinska Institutet, Stockholm, Sweden

399. Department of Numerical Analysis and Computer Science, SciLifeLab, Stockholm University, Stockholm, Sweden

400. Department of Public Health and Primary Care, Strangeways Research Laboratory, University of Cambridge, Cambridge, UK

401. Division of Nephrology, Department of Internal Medicine and Medical Specialties, Columbus-Gemelli University Hospital, Catholic University, Rome, Italy

402. Department of Cardiovascular Medicine, Wellcome Trust Centre for Human Genetics, University of Oxford, Oxford, UK

403. University of Iceland, 101 Reykjavik, Iceland

404. Department of Biobank Research, Umeå University, Umeå, Sweden

405. MRC Human Genetics Unit, MRC IGMM, University of Edinburgh, Edinburgh, Scotland

406. Department of Biomedical Technology, Sangmyung University, Chungnam, Korea

407. Section on Statistical Genetics, Department of Biostatistics, University of Alabama at Birmingham, Birmingham, AL, USA

408. Memory Aging and Cognition Centre (MACC), National University Health System, Singapore

409. Echinos Medical Centre, Echinos, Greece

410. Division of Research, Kaiser Permanente, Northern California Region, Oakland, CA, USA

411. Division of Human Genetics, Genome Institute of Singapore, Singapore

412. Fairbanks School of Public Health, Indiana University, Indianapolis, IN, USA

413. Division of Endocrine and Metabolism, Department of Internal Medicine, Taichung Veterans General Hospital, Taichung, Taiwan

414. Department of Medical Research, Taichung Veterans General Hospital, Taichung, Taiwan

415. Department of Epidemiology, Johns Hopkins University, Baltimore, MD, USA

416. Department of Medicine, Johns Hopkins University, Baltimore, MD, USA

417. Center for Molecular Medicine and Genetics, Wayne State University, Detroit, MI, USA

418. Department of Neurology, Wayne State University School of Medicine, Detroit, MI, USA

419. Division of Cardiology, Brigham and Women's Hospital, Boston, MA, USA

420. Division of Cardiology, Harvard Medical School, Boston, MA, USA

421. National and Kapodistrian University of Athens, Dromokaiteio Psychiatric Hospital, Athens, Greece

422. University of Athens, Department of Dietetics and Nutritional Science, Harokopio University, Athens, Greece

423. Division of General Internal Medicine, Johns Hopkins University School of Medicine, Baltimore, MD, USA

424. Welch Center for Prevention, Epidemiology, and Clinical Research, Johns Hopkins University, Baltimore, MD, USA

425. USC Eye Institute, Department of Ophthalmology, Keck School of Medicine of the University of Southern California, Los Angeles, CA, USA

426. Department of Molecular Epidemiology, German Institute of Human Nutrition Potsdam-RehbruÌĹcke, Nuthetal, Germany

427. Department of Epidemiology, Colorado School of Public Health, University of Colorado Denver, Aurora, CO, USA

428. Department of Nutrition and Dietetics, Harokopio University, Athens, Greece

429. University of Cambridge Metabolic Research Laboratories, MRC Metabolic Diseases Unit and NIHR Cambridge Biomedical Research Centre, Wellcome Trust-MRC Institute of Metabolic Science, Addenbrooke's Hospital, Cambridge, UK

430. Department of Internal Medicine, Division of Endocrinology, Metabolism, Pathobiochemistry and Clinical Chemistry and Institute of Diabetes Research and Metabolic Diseases, University of TuÌĹbingen, TuÌĹbingen, Germany

431. German Center for Diabetes Research (DZD), Germany

432. The Netherlands Genomics Initiative-sponsored Netherlands Consortium for Healthy Aging (NGI-NCHA), Leiden/Rotterdam, the Netherlands

433. Department of Public Health, Faculty of Medicine, University of Split, Split, Croatia

434. The Oxford Centre for Diabetes, Endocrinology and Metabolism, University of Oxford, Oxford, UK

435. Wellcome Trust Centre for Human Genetics, Oxford, UK

436. Centre for Population Health Sciences, Medical School, University of Edinburgh, Edinburgh, Scotland

437. Generation Scotland, A Collaboration between the University Medical Schools and NHS, Aberdeen, Dundee, Edinburgh, and Glasgow, UK

438. Science for Life Laboratory, Karolinska Institutet, Stockholm, Sweden

439. Department of Gastroenterology, Gennimatas General Hospital, Athens, Greece

440. College of Medicine, National Defense Medical Center, Taipei, Taiwan

441. School of Medicine, National Yang-Ming University, Taipei, Taiwan

442. Department of Epidemiology, School of Public Health, University of Michigan, Ann Arbor, MI, USA

443. Medical Research Institute, University of Dundee, Dundee, UK

444. Department of Hematology, Long Road, Cambridge, UK

445. Department of Internal Medicine, Division of Gastroenterology and Department of Computational Medicine and Bioinformatics, University of Michigan, Ann Arbor, MI, USA

446. Division of Psychiatric Genomics, Icahn School of Medicine at Mount Sinai, New York, NY, USA

447. First Department of Propaedeutic and Internal Medicine, Athens University Medical School, Laiko General Hospital, Athens, Greece

448. Diabetes Centre, 2nd Department of Internal Medicine, National University of Athens, Hippokration General Hospital, Athens, Greece

449. Anogia Medical Centre, Anogia, Greece

450. Center for Medical Systems Biology, Leiden, The Netherlands

451. Department of Clinical Sciences, Genetic and Molecular Epidemiology Unit, Lund University, Skåne University Hospital, Malmö, Sweden

452. Division of Public Health Sciences, Wake Forest School of Medicine, Winston-Salem, NC, USA

453. Institute of Cellular Medicine, Newcastle University, Newcastle-upon-Tyne, UK

454. Dromokaiteio Psychiatric Hospital, Athens, Greece

455. University of Sheffield, Sheffield, UK

Extended Acknowledgements

This work was supported by NIH/NIDDK U01 DK105554 to JCF. This research has been conducted using the UK Biobank Resource under application number 27892. MSU is supported by NIH/NIDDK K23 DK114551. AODL was supported by NIH/NICHD K12 HD052896. MB is supported by NIH/NIDDK DK062370. JCF is also supported by NIH/NIDDK K24 DK110550. JMM is supported by American Diabetes Association Innovative and Clinical Translational Award 1-19-ICTS-068.

Funding for GO ESP was provided by NHLBI grants RC2 HL-103010 (HeartGO), with exome sequencing was performed through NHLBI grants RC2 HL-102925 (BroadGO) and RC2 HL-102926 (SeattleGO). HeartGO components and their support include Atherosclerosis Risk in Communities (NHLBI contracts N01 HC-55015, N01 HC-55016, N01HC-55017, N01 HC-55018, N01 HC-55019, N01 HC-55020, and N01 HC-55021); Cardiovascular Health Study (NHLBI contracts HHSN268201200036C, HHSN268200800007C, N01HC55222, N01HC85079, N01HC85080, N01HC85081, N01HC85082, N01HC85083, and N01HC85086); and NHLBI grants U01HL080295, R01HL087652, R01HL105756, R01HL103612, and R01HL120393, with additional contribution from the National Institute of Neurological Disorders and Stroke. Additional support was provided through R01AG023629 from the National Institute on Aging. A full list of principal Cardiovascular Health Study investigators and institutions can be found at CHS-NHLBI.org; Coronary Artery Risk Development in Young Adults (NHLBI contracts N01-HC95095, N01-HC48047, N01-HC48048, N01-HC48049, and N01-HC48050); Framingham Heart Study (NHLBI contract N01-HC-25195 and grants NS17950, AG08122, and AG033193); Jackson Heart Study (NHLBI contracts N01 HC-95170, N01 HC-95171, and N01 HC-95172). MESA project is conducted and supported by the National Heart, Lung, and Blood Institute (NHLBI) in collaboration with MESA investigators. Support for MESA is provided by contracts 75N92020D00001, HHSN268201500003I, N01-HC-95159, 75N92020D00005, N01-HC-95160, 75N92020D00002, N01-HC-95161, 75N92020D00003, N01-HC-95162, 75N92020D00006, N01-HC-95163, 75N92020D00004, N01-HC-95164, 75N92020D00007, N01-HC-95165, N01-HC-95166, N01-HC-95167, N01-HC-95168, N01-HC-95169, UL1-TR-000040, UL1-TR-001079, UL1-TR-001420 . This study was also supported in part by the National Center for Advancing Translational Sciences, CTSI grant UL1TR001881, the National Institute of Diabetes and Digestive and Kidney Disease Diabetes Research Center (DRC) grant DK063491 to the Southern California Diabetes Endocrinology Research Center.

Cardiovascular Health Study: This CHS research was supported by NHLBI contracts HHSN268201200036C, HHSN268200800007C, HHSN268201800001C, N01HC55222, N01HC85079, N01HC85080, N01HC85081, N01HC85082, N01HC85083, N01HC85086; and NHLBI grants U01HL080295, R01HL087652, R01HL105756, R01HL103612, R01HL120393, and U01HL130114 with additional contribution from the National Institute of Neurological Disorders and Stroke (NINDS). Additional support was provided through R01AG023629 from the National Institute on Aging (NIA). A full list of principal CHS investigators and institutions can be found at CHS-NHLBI.org. We gratefully acknowledge the Eunice Kennedy National Institute of Child Health and Human Development for support of the MODY variant curation through U24

HD093486 (to TIP). The content is solely the responsibility of the authors and does not necessarily represent the official views of the National Institutes of Health.

The Jackson Heart Study (JHS) is supported and conducted in collaboration with Jackson State University (HHSN268201800013I), Tougaloo College (HHSN268201800014I), the Mississippi State Department of Health (HHSN268201800015I) and the University of Mississippi Medical Center (HHSN268201800010I, HHSN268201800011I and HHSN268201800012I) contracts from the National Heart, Lung, and Blood Institute (NHLBI) and the National Institute on Minority Health and Health Disparities (NIMHD). The authors also wish to thank the staffs and participants of the JHS.

Novo Nordisk Foundation Center for Basic Metabolic Research is an independent Research Center, based at the University of Copenhagen, Denmark and partially funded by an unconditional donation from the Novo Nordisk Foundation (www.cbmr.ku.dk) (Grant number NNF18CC0034900).

The LOLIPOP study is supported by the National Institute for Health Research (NIHR) Comprehensive Biomedical Research Centre Imperial College Healthcare NHS Trust, the NIHR Official Development Assistance (ODA, award 16/136/68), the European Union FP7 (EpiMigrant, 279143) and H2020 programs (iHealth-T2D, 643774). The views expressed are those of the author(s) and not necessarily those of the Imperial College Healthcare NHS Trust, the NHS, the NIHR or the Department of Health. We thank the participants and research staff who made the study possible. JC is supported by the Singapore Ministry of Health's National Medical Research Council under its Singapore Translational Research Investigator (STaR) Award (NMRC/STaR/0028/2017).

The TwinsUK study was funded by the Wellcome Trust and European Community's Seventh Framework Programme (FP7/2007-2013). The TwinsUK study also receives support from the National Institute for Health Research (NIHR)- funded BioResource, Clinical Research Facility and Biomedical Research Centre based at Guy's and St Thomas' NHS Foundation Trust in partnership with King's College London.

The views expressed in this article are those of the author(s) and not necessarily those of the NHS, the NIHR, or the Department of Health. MMcC has served on advisory panels for Pfizer, NovoNordisk and Zoe Global, has received honoraria from Merck, Pfizer, Novo Nordisk and Eli Lilly, and research funding from Abbvie, Astra Zeneca, Boehringer Ingelheim, Eli Lilly, Janssen, Merck, NovoNordisk, Pfizer, Roche, Sanofi Aventis, Servier, and Takeda. As of June 2019, MMcC is an employee of Genentech, and a holder of Roche stock. MMcC wishes to acknowledge NIDDK U01-DK105535 and Wellcome: 090532, 098381, 106130, 203141, 212259.

The San Antonio Mexican American Family Studies (SAMAFS) are supported by the following grants/institutes. The San Antonio Family Heart Study (SAFHS) and San Antonio Family Diabetes/Gallbladder Study (SAFDGS) were supported by U01DK085524, R01 HL0113323,

P01 HL045222, R01 DK047482 and R01 DK053889. The Veterans Administration Genetic Epidemiology Study (VAGES) study was supported by a Veterans Administration Epidemiologic grant. The Family Investigation of Nephropathy and Diabetes - San Antonio (FIND-SA) study was supported by NIH grant U01DK57295. The SAMAFS research team acknowledges the contributions of late Dr. H. E. Abboud to the research activities of the SAMAFS.

The KARE cohort was supported by grants from Korea Centers for Disease Control and Prevention(4845–301, 4851–302, 4851–307) and intramural grants from the Korea National Institute of Health (2016-NI73001-00, 2019-NG-053-01).

RJFL is supported by the NIH (R01DK110113, R01DK107786, 1R01DK124097). NC is supported by a grant from the Canadian Institutes of Health Research (CIHR Fellowship). The Mount Sinai BioMe Biobank has been supported by The Andrea and Charles Bronfman Philanthropies and in part by Federal funds from the NHLBI and NHGRI (U01HG00638001; U01HG007417; X01HL134588).

The Framingham Heart Study (FHS) acknowledges the support of Contracts NO1-HC-25195, HHSN268201500001I and 75N92019D00031 from the National Heart, Lung and Blood Institute and grant supplement R01 HL092577-06S1 for this research. We also acknowledge the dedication of the FHS study participants without whom this research would not be possible. Dr. Vasan is supported in part by the Evans Medical Foundation and the Jay and Louis Coffman Endowment from the Department of Medicine, Boston University School of Medicine.
