# Supplementary material for: Determinants of penetrance and variable expressivity in monogenic metabolic conditions across 77,184 exomes
Source: Nat Commun. 2021 Jun 9;12:3505. doi: 10.1038/s41467-021-23556-4 (PMC8190084; doi:10.1038/s41467-021-23556-4)
Supplement: Supplementary file 2 — Description of Additional Supplementary Files [file 41467_2021_23556_MOESM2_ESM.docx]

Descriptions of Additional Supplementary Files

**Supplementary Data 1**

**Description:** Detailed characteristics of study cohorts. AMP-T2D-GENES Cohort Information (adapted from Flannick et al, 2019 Supplementary Table 1).

**Supplementary Data 2**

**Description:** Variant curation assessments and carrier counts.

**Supplementary Data 3**

**Description:** Effect size and penetrance estimates of clinically significant monogenic variants across conditions and by gene.

**Supplementary Data 4**

**Description:** Effect size and penetrance in filtered out variants (designated benign, likely benign, or not pLOF).

**Supplementary Data 5**

**Description:** Clinical characteristics of monogenic diabetes variant carriers.

**Supplementary Data 6**

**Description:** pLoF curation categories and guidelines for classification.
