# Supplementary material for: Determinants of penetrance and variable expressivity in monogenic metabolic conditions across 77,184 exomes
Source: Nat Commun. 2021 Jun 9;12:3505. doi: 10.1038/s41467-021-23556-4 (PMC8190084; doi:10.1038/s41467-021-23556-4)
Supplement: Supplementary file 4 — Reporting Summary [file 41467_2021_23556_MOESM4_ESM.pdf]

nature research

Corresponding author(s): Miriam S. Udler

Last updated by author(s): Mar 30, 2021

Reporting Summary

Nature Research wishes to improve the reproducibility of the work that we publish. This form provides structure for consistency and transparency in reporting. For further information on Nature Research policies, see our Editorial Policies and the Editorial Policy Checklist.

Statistics

For all statistical analyses, confirm that the following items are present in the figure legend, table legend, main text, or Methods section.

n/a	Confirmed	
☐	☒	The exact sample size (n) for each experimental group/condition, given as a discrete number and unit of measurement
☐	☒	A statement on whether measurements were taken from distinct samples or whether the same sample was measured repeatedly
☐	☒	The statistical test(s) used AND whether they are one- or two-sided<br>*Only common tests should be described solely by name; describe more complex techniques in the Methods section.*
☐	☒	A description of all covariates tested
☐	☒	A description of any assumptions or corrections, such as tests of normality and adjustment for multiple comparisons
☐	☒	A full description of the statistical parameters including central tendency (e.g. means) or other basic estimates (e.g. regression coefficient) AND variation (e.g. standard deviation) or associated estimates of uncertainty (e.g. confidence intervals)
☐	☒	For null hypothesis testing, the test statistic (e.g. F, t, r) with confidence intervals, effect sizes, degrees of freedom and P value noted<br>*Give P values as exact values whenever suitable.*
☒	☐	For Bayesian analysis, information on the choice of priors and Markov chain Monte Carlo settings
☒	☐	For hierarchical and complex designs, identification of the appropriate level for tests and full reporting of outcomes
☐	☒	Estimates of effect sizes (e.g. Cohen's d, Pearson's r), indicating how they were calculated

Our web collection on statistics for biologists contains articles on many of the points above.

Software and code

Policy information about availability of computer code

Data collection	No software was used in data collection.
Data analysis	All software used in the analysis were open source and described in the Methods section of the manuscript. Existing software packages used were: Plink 1.9, EPACTS v3.2.4, Rv3.5.2, Hail v0.2.54, Alamut v2.11, LDpred v1.0.6, Ensembl's Variant Effect Predictor (VEP) versions 85 and 95, Aberrant v1.0 R package, and LOFTEE. Code written for analyses performed in the manuscript are available in GitHub: https://github.com/broadinstitute/exome_penetrance.

For manuscripts utilizing custom algorithms or software that are central to the research but not yet described in published literature, software must be made available to editors and reviewers. We strongly encourage code deposition in a community repository (e.g. GitHub). See the Nature Research guidelines for submitting code & software for further information.

Data

Policy information about availability of data

All manuscripts must include a data availability statement. This statement should provide the following information, where applicable:
- Accession codes, unique identifiers, or web links for publicly available datasets
- A list of figures that have associated raw data
- A description of any restrictions on data availability

Sequence data and phenotypes for this study from the AMP-T2D-GENES study are available via the database of Genotypes and Phenotypes (dbGAP) and/or the European Genome-phenome Archive, as indicated in Supplementary Table 2. Access to data from the UK Biobank can be obtained at https://www.ukbiobank.ac.uk/enable-your-research.All variants curated for this project, along with their classification and supporting evidence, were submitted to the ClinVar database (https://www.ncbi.nlm.nih.gov/clinvar/) on January 30th, 2020.

The following databases were accessed for this work: ClinVar (https://www.ncbi.nlm.nih.gov/clinvar/), Human Gene Mutation Database (https://digitalinsights.qiagen.com/products-overview/clinical-insights-portfolio/human-gene-mutation-database/), gnomAD v2.1.1 (https://gnomad.broadinstitute.org/), PubMed (https://pubmed.ncbi.nlm.nih.gov/), Google Scholar (https://scholar.google.com/), Alamut v.2.11 (https://www.interactive-biosoftware.com/alamut-visual/), and the UCSC browser (https://genome.ucsc.edu/).

Field-specific reporting

Please select the one below that is the best fit for your research. If you are not sure, read the appropriate sections before making your selection.

[x] Life sciences　　[ ] Behavioural & social sciences　　[ ] Ecological, evolutionary & environmental sciences

For a reference copy of the document with all sections, see nature.com/documents/nr-reporting-summary-flat.pdf

Life sciences study design

All studies must disclose on these points even when the disclosure is negative.

Sample size	The total sample size was 77,184 adult individuals, which included 38,618 multi-ancestral individuals from a type 2 diabetes case-control study AMP-T2D-GENES and 38,566 individuals from UK Biobank. The sample size was the largest possible dataset we could access with both genetic sequence data and phenotypic data available.
Data exclusions	To provide consistency between the two datasets analyzed, individuals in the AMP-T2D-GENES dataset younger than age 40 were excluded. Individuals recruited to the Pakistan Genomic Resource cohort were also excluded for all analyses involving lipid levels or BMI at the request of the study's PI. Additionally, both datasets were restricted to unrelated individuals. For analyses involving polygenic scores, only UK Biobank had both the required exome and SNP data, and the overwhelming majority of these individuals were of European ancestry, so we restricted analyses in the UK Biobank to this subset of individuals of European ancestry.
Replication	Analyses of monogenic variant carriers were replicated using two separate cohorts: the AMP-T2D-GENES dataset of type 2 diabetes cases and controls, and the UK Biobank population based study. The effect of monogenic variants on biomarkers were remarkably consistent across the two studies, but estimates of penetrance showed more variability, reflecting different environmental and/or genetic factors contributing to the metabolic conditions we studied.
Randomization	This study did not involve randomization. Group allocations were based on monogenic variant carrier status. The covariates that were used included age, sex, the first ten principal components, and sequencing technology (described in more details in Methods).
Blinding	Analysts who performed the curation of the genetic variants were blinded to phenotypic information for the participant. There was no other blinding as part of this study.

Reporting for specific materials, systems and methods

We require information from authors about some types of materials, experimental systems and methods used in many studies. Here, indicate whether each material, system or method listed is relevant to your study. If you are not sure if a list item applies to your research, read the appropriate section before selecting a response.

Materials & experimental systems

n/a	Involved in the study	
[x]	[ ]	Antibodies
[x]	[ ]	Eukaryotic cell lines
[x]	[ ]	Palaeontology and archaeology
[x]	[ ]	Animals and other organisms
[ ]	[x]	Human research participants
[x]	[ ]	Clinical data
[x]	[ ]	Dual use research of concern

Methods

n/a	Involved in the study	
[x]	[ ]	ChIP-seq
[x]	[ ]	Flow cytometry
[x]	[ ]	MRI-based neuroimaging

Human research participants

Policy information about studies involving human research participants

Population characteristics	The complete AMP-T2D-GENES cohort consists of 20,791 cases and 24,440 controls selected from multiple distinct multi-ancestry studies. The present study includes a subset of 22,875 with type 2 diabetes or prediabetes and 15,743 controls, all age 40 or older, from studies who consented for the data to be used in this analysis. Individuals had data available for exome sequences and phenotypes related to glycemia, lipid traits, and body mass index. They were 48% male and the mean age was 58 in cases, 57 in controls, and mean bmi was 29 in cases, 27 in controls.<br><br>UK Biobank is a prospective cohort of approximately 500,000 recruited individuals from the general population aged 40–69 years in 2006–2010 from across the United Kingdom, with genotype, phenotype, and linked healthcare record data. Our analysis focused on the subset of 38,566 individuals who had data available for exome sequences and phenotypes related to glycemia, lipid traits, and body mass index. Within this subset, 46% were male, the mean age was 58, and the mean BMI was 27.
Recruitment	Recruitment strategies differed within the 26 sub-studies that make up AMP-T2D-GENES and are detailed in Supplementary Data 1. Participants in AMP-T2D-GENES were selected to be type 2 diabetes cases (or controls), and specific exclusion practices were employed by several studies to remove possible monogenic diabetes cases based on expected clinical features; this recruitment strategy could have resulted in the individuals we identified as having monogenic diabetes being less likely to have classic features of the condition. The UK Biobank is a population-based study that recruited volunteers and is known to have a "healthy participant" bias which would mean that estimates of penetrance would be biased downward. The potential biases in both datasets are discussed in the manuscript.
Ethics oversight	All samples were approved for use by their home institution's institutional review board or ethics committee. Samples newly sequenced at The Broad Institute as part of T2D-GENES and SIGMA, as well as utilization of UK Biobank data, are covered under Partners Human Research Committee protocol # 2017P000445/PHS "Diabetes Genetics and Related Traits".

Note that full information on the approval of the study protocol must also be provided in the manuscript.
